# C-X-C domain ligand 14-mediated stromal cell–macrophage interaction as a therapeutic target for hand dermal fibrosis

**DOI:** 10.1038/s42003-023-05558-8

**Published:** 2023-11-18

**Authors:** Atsushi Goto, Shingo Komura, Koki Kato, Rie Maki, Akihiro Hirakawa, Hiroyuki Tomita, Akihiro Hirata, Yasuhiro Yamada, Haruhiko Akiyama

**Affiliations:** 1 Department of Orthopaedic Surgery, Gifu University Graduate School of Medicine, Gifu, 501-1194 Japan; 2 Department of Tumor Pathology, Gifu University Graduate School of Medicine, Gifu, 501-1194 Japan; 3 Laboratory of Veterinary Pathology, Joint Department of Veterinary Medicine, Faculty of Applied Biological Sciences, Gifu University, Gifu, 501-1194 Japan; 4 Department of Molecular Pathology, Graduate School of Medicine, The University of Tokyo, Tokyo, 113-0033 Japan

**Keywords:** Mechanisms of disease, Chemotaxis

## Abstract

Dupuytren’s contracture, a superficial dermal fibrosis, causes flexion contracture of the affected finger, impairing hand function. Specific single-nucleotide polymorphisms within genes in the Wnt signalling pathway are associated with the disease. However, the precise role of Wnt signalling dysregulation in the onset and progression of Dupuytren’s contracture remains unclear. Here, using a fibrosis mouse model and clinical samples of human Dupuytren’s contractures, we demonstrate that the activation of Wnt/β-catenin signalling in *Tppp3*-positive cells in the dermis of the paw is associated with the development of fibrosis. Fibrosis development and progression via Wnt/β-catenin signalling are closely related to stromal cell–macrophage interactions, and Wnt/β-catenin signalling activation in *Tppp3*-positive stromal cells causes M2 macrophage infiltration via chemokine Cxcl14, resulting in the formation of a TGF-β-expressing fibrotic niche. Inhibition of Cxcl14 mitigates fibrosis by decreasing macrophage infiltration. These findings suggest that *Cxcl14*-mediated stromal cell–macrophage interaction is a promising therapeutic target for Wnt/β-catenin-induced fibrosis.

## Abstract (web summary)

Wnt/β-catenin signalling activation in the dermis cause fibrosis by cell-autonomous program and by a non-cell-autonomous program mediated by TGF-β niche formation based on Cxcl14-mediated interactions between stromal cells and M2 macrophages.

## Introduction

Dupuytren’s contracture is a superficial dermal fibrotic disease in the hand that causes flexion contracture of the affected fingers and impairs hand function. Genetic predisposition, ethnic characteristics, and other factors, including alcohol intake, smoking, diabetes, repetitive trauma, and exposure to vibration, have been identified as risk factors for Dupuytren’s contracture development, suggesting that it is a multifactorial disease^1^. Dupuytren’s contracture represents a prevalent condition, particularly among Western populations, with an estimated prevalence of 21% by the age of 65 years^2^. Although a collagenase *Clostridium histolyticum* injection is available as a minimally invasive therapy, surgical excision remains a reliable treatment method for this disease^3^. However, surgical complications, including hematomas, nerve injuries, and wound healing complications, are frequently observed, with incidences of 23%^4^.

Myofibroblasts, the predominant stromal cells in Dupuytren’s contracture, play a central role in fibrosis by producing excessive extracellular matrix and pathological collagen^5^. Recent genome-wide studies have identified single-nucleotide polymorphisms (SNPs) in a subset of genes involved in the Wnt signalling pathway, including *WNT2, WNT4, WNT7B, SFRP4, RSPO2*, and *SULF1*^6,7^. β-catenin, a downstream target of canonical Wnt signalling, is expressed in most myofibroblasts in the nodule, accumulating in their nuclei^8^. Therefore, the involvement of such Wnt signalling-related SNPs in Dupuytren’s contracture has been investigated^7,9–11^. The expression of *WNT7B*, a canonical Wnt ligand, was increased in Dupuytren’s nodules compared with that in unaffected palmar aponeurosis^9,10^. Moreover, its risk genotype was associated with Dupuytren’s contracture development^11^. The mRNA expression of *SFRP4*, a Wnt antagonist, was increased in nodules compared with that in unaffected palmar aponeurosis in Dupuytren’s patients^7,9^. However, its protein secretion decreased in Dupuytren’s tissues with a high-risk genotype compared with that in those with a low-risk genotype^7^. Thus, the Wnt signalling pathway is likely implicated in Dupuytren’s contracture.

Recent studies revealed the possible involvement of stromal cell–immune cell interaction in myofibroblast differentiation and its activation in Dupuytren’s contracture^12–14^. Mast cells and macrophages, especially alternatively activated macrophages (M2), are involved in the fibrotic mechanism via chemotactic factors^12–14^. However, the role of Wnt signalling activation in the development and progression of Dupuytren’s contracture remains to be elucidated. Moreover, additional research is required to investigate the role of Wnt signalling in the interaction between stromal and immune cells.

The aim of this study was to investigate the relationship between Wnt signalling activation and the development of dermal fibrosis using a fibrosis mouse model and human clinical samples of Dupuytren’s contractures. Our analyses revealed that activation of Wnt/β-catenin signalling in dermal tubulin polymerization-promoting protein family member 3 (*Tppp3*)-positive cells resulted in the development of fibrosis of the paw, and its progression was markedly dependent on Wnt/β-catenin signalling activity. However, in addition to Wnt/β-catenin signalling-dependent myofibroblast differentiation in a cell-autonomous manner, fibrosis development and progression via Wnt/β-catenin signalling were closely related to stromal cell-immune cell interactions marked by M2 macrophage infiltration. Furthermore, transforming growth factor-β (TGF-β) was highly expressed in the fibrotic niche, consisting of stromal cells and macrophages, causing myofibroblast differentiation of stromal cells. Notably, the activation of Wnt/β-catenin signalling in *Tppp3*-positive cells upregulated the expression of chemokine C-X-C domain ligand 14 (*Cxcl14*), which is involved in macrophage infiltration. Furthermore, the blockade of Cxcl14 mitigated Wnt/β-catenin-induced fibrosis of the paw by decreasing macrophage infiltration.

## Results

### TPPP3 is expressed in the human palmar fascia and Dupuytren’s contractures

A recent study analysed human clinical samples and reported that pericytes are the putative cellular origin of Dupuytren’s contractures^15^; however, their exact origin remains unknown^1^. Therefore, we investigated clinical samples of the human palmar fascia and Dupuytren’s contracture to identify candidate markers of origin cells and establish a dermal fibrosis mouse model that mimics Dupuytren’s contractures.

**Fig. 1:**
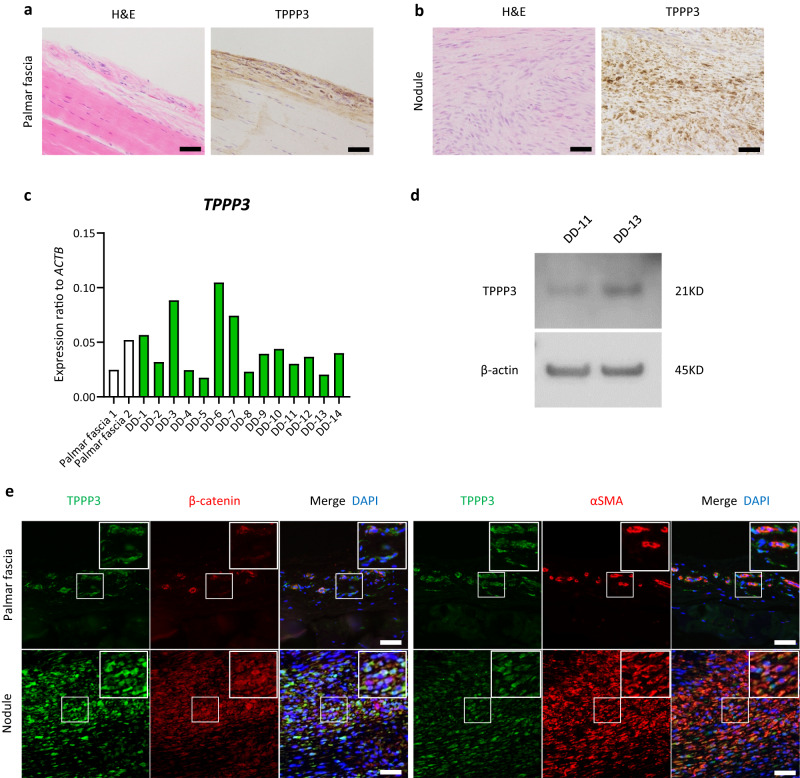
TPPP3 is expressed in the human palmar fascia and Dupuytren’s contractures. **a** Haematoxylin and eosin staining (H&E) (left) and immunohistochemistry (right) of the human palmar fascia. TPPP3 expression was detectable in the peripheral connective tissue around the collagenous palmar fascia. **b** H&E staining (left) and immunohistochemistry (right) of human Dupuytren’s contracture tissues. TPPP3 was strongly expressed in diseased cells in the nodule. **c** RT-qPCR results show the expression level of *TPPP3* in 14 freshly isolated nodules of human Dupuytren’s contractures (DD-1 to DD-14) (also see Supplementary Fig. 1) and in unaffected palmar fascia. *TPPP3* expression was normalized to *ACTB* expression. The data are presented as the mean of three technical replicates. **d** Detection of TPPP3 expression in human Dupuytren’s contracture samples (DD-11 and DD-13) using Western blotting. **e** Fluorescent immunohistochemistry of human palmar fascia (upper) and Dupuytren’s contracture tissues (lower). TPPP3-expressing cells with fibroblast-like morphology on the surface of the palmar fascia did not co-express β-catenin or αSMA. Conversely, some TPPP3-expressing cells with vessel-like morphology co-expressed β-catenin and αSMA (upper). Most TPPP3-expressing cells in the nodule co-expressed β-catenin (upper) and the myofibroblast marker αSMA (lower). The white square area is enlarged in the upper right panel. Scale bars represent 50 µm (**a**, **b**, **d**).

Dupuytren’s contractures generally occur in the dermis of the palmar hand and continue to the palmar fascia. *TPPP3* is a known paratenon and synovial cell marker around the tendon and fascia^16,17^. As expected, TPPP3 expression was detected in the cells on the surface of the palmar fascia (Fig. 1a). In addition, TPPP3 was expressed in human Dupuytren’s contractures, with strong expression in the nodule (Fig. 1b–d, Supplementary Fig. 1). Immunohistochemical analyses revealed that *TPPP3*-positive cells with fibroblast-like morphology on the surface of the palmar fascia did not co-express β-catenin and αSMA. Conversely, most *TPPP3*-positive cells in the nodule co-expressed β-catenin and αSMA, a myofibroblast marker (Fig. 1e). These results suggest that *TPPP3*-positive palmar fascial cells in the dermis potentially represent one of the candidates for cellular origin of Dupuytren’s contractures.

### Activation of Wnt/β-catenin signalling in *Tppp3*-positive cells causes dermal fibrosis of the paws

**Fig. 2:**
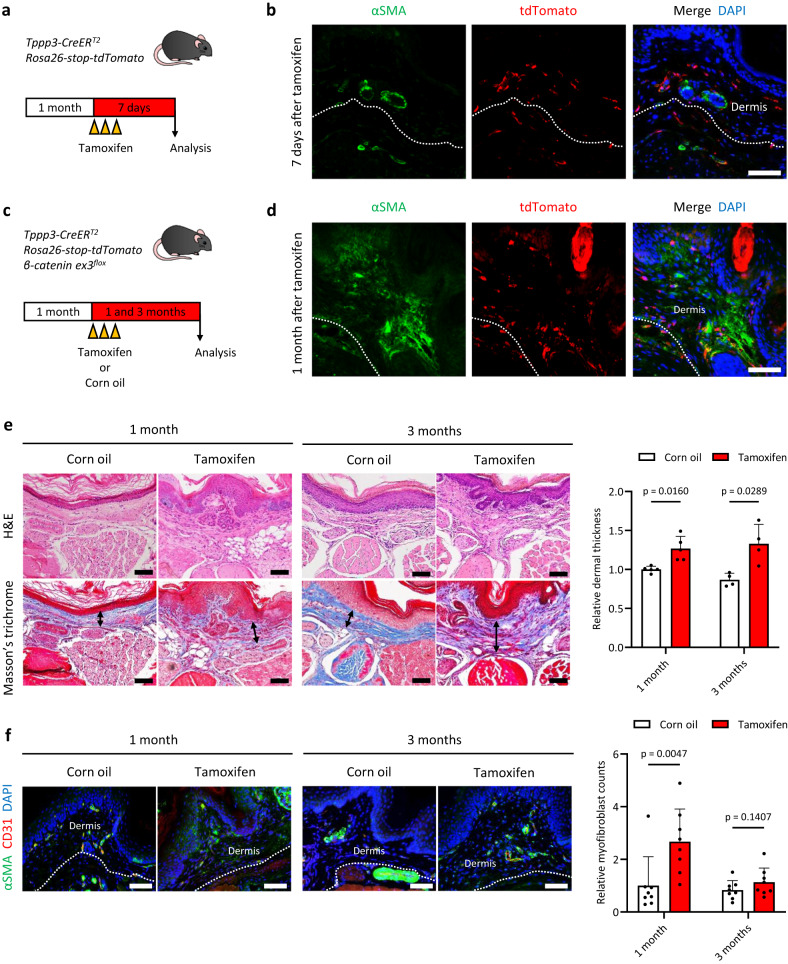
Wnt signalling activation in *Tppp3*-expressing cells causes dermal fibrosis of the paw. **a** A schematic of the tamoxifen injection protocol for *Tppp3-CreER*^*T2*^*/Rosa26-stop-tdTomato(R26-tdT)* mice. At 1 month of age, the mice were injected with 1 mg tamoxifen three times and analysed 7 days after the last injection. **b** Fluorescent immunohistochemistry of αSMA (green) and tdTomato (red) in the palmar side of the paw in *Tppp3-CreER*^*T2*^*/Rosa26-tdT* mice. Several tdTomato-positive cells existed in the dermal layer of the paw. Dotted lines indicate the border of the dermis. **c** A schematic representation of the in vivo experimental setup involving *Tppp3-CreER*^*T2*^*/R26-tdT/β-catenin ex3* ^*flox*^ mice. Notably, activated β-catenin without phosphorylation sites in exon 3 can be induced in *Tppp3*-positive cells via tamoxifen injection. At 1 month of age, the mice were injected with 1 mg of tamoxifen or corn oil (control) and analysed 1 and 3 months after the injections. **d** Fluorescent immunohistochemistry of murine dermal fibrotic lesions in the paw 1 month after tamoxifen injections. β-catenin activation in the dermal *Tppp3*-lineage cells (tdTomato^+^) caused fibrosis, as indicated by αSMA expression. Dotted lines indicate the border of the dermis. **e** H&E staining and Masson’s trichrome staining of *Tppp3-CreER*^*T2*^*/R26-tdT/β-catenin ex3* ^*flox*^ mice treated with corn oil or tamoxifen 1 and 3 months after injections. Compared to the corn oil groups (left panels), the tamoxifen groups (right panels) exhibited a notable and statistically significant increase in dermal thickness on the palmar side of the paw at both 1 and 3 months (*p* = 0.0160 and 0.0289, respectively). The graph illustrates the relative dermal thickness, with the mean dermal thickness in each sample of the corn-oil-treated group at 1 month set to 1. The mean ± SD of five and four independent biological samples (six different lesions per sample) are shown (two-tailed Mann–Whitney U test). Arrows in Masson’s trichrome staining indicate the thickness of the dermis. **f** Fluorescent immunohistochemistry of the palmar side of the paws in *Tppp3-CreER*^*T2*^*/R26-tdT/β-catenin ex3* ^*flox*^ mice treated with corn oil (lefts) or tamoxifen (rights) 1 and 3 months after injections. Myofibroblasts were defined as αSMA (green)-positive/CD31 (red)-negative cells. The tamoxifen group showed significantly increased dermal myofibroblasts in the palmar side of the paw at 1 month (*p* = 0.0047); at 3 months, the increase was not significant (*p* = 0.1407). The graph indicates the relative number of myofibroblasts per area. The mean count of myofibroblasts within each sample from the corn oil treatment group at 1 month was set to 1. The mean ± SD of four independent biological samples (two different lesions per sample) are shown (two-tailed Mann–Whitney U test). Scale bars represent 50 µm (**b**, **d**, **e**, **f**).

We investigated the localization of *Tppp3*-positive cells in the paws using tamoxifen-administered *Tppp3-CreER*^*T2*^*/Rosa26-loxP-stop-loxP-tdTomato* (*Rosa26-stop-tdTomato*) mice and confirmed that many tdTomato-positive cells were localized in the dermis of the paw (Fig. 2a, b). We created *Tppp3-CreER*^*T2*^*/Rosa26-stop-tdTomato/β-catenin ex3*^*flox*^ mice and induced Wnt/β-catenin signalling activation in *Tppp3*-positive cells (Fig. 2c, Supplementary Fig. 2a–d). After tamoxifen administration, fibrotic regions comprising αSMA-expressing myofibroblasts were induced in the paw; however, it is noteworthy that the mice did not develop nodules and finger contractures analogous to those observed in human Dupuytren’s contractures (Fig. 2d). Sequential histological analyses revealed that the dermal layer was thickened in a time-dependent manner, although they were not palmar side-specific (Fig. 2e, Supplementary Fig. 2e). Dermal fibrotic region exhibited a significant increase in the number of myofibroblasts one month after Wnt/β-catenin signalling activation, followed by a subsequent reduction two months thereafter (Fig. 2f).

### Wnt/β-catenin signalling regulates myofibroblast phenotype and activity

**Fig. 3:**
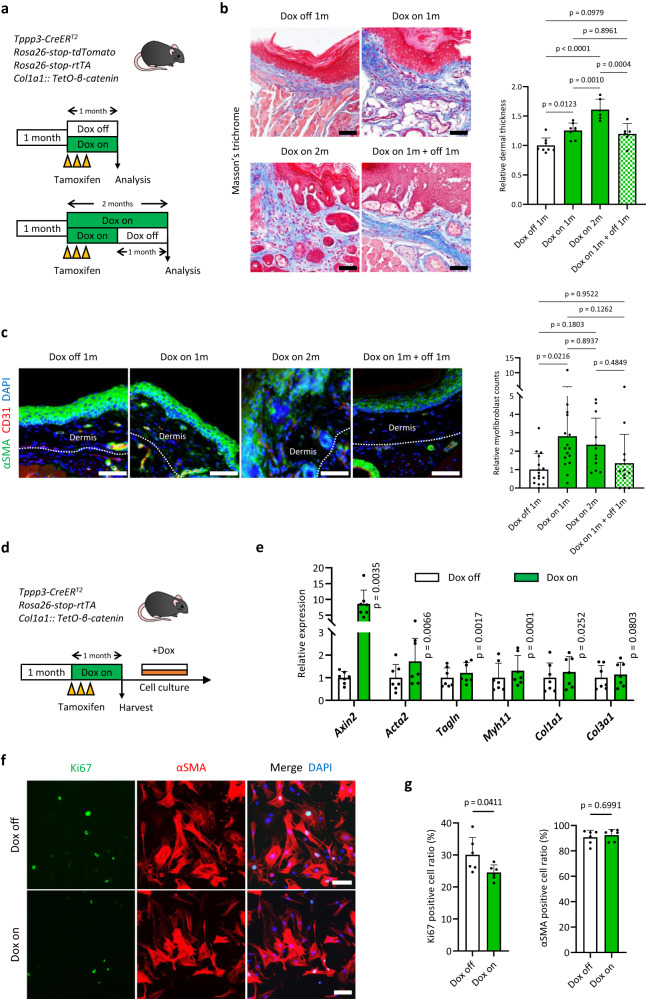
The development and progression of dermal fibrosis in mice are dependent on Wnt signalling activity. **a** A schematic representation of the in vivo experiment conducted on doxycycline (Dox)-inducible β-catenin-expressing mice (*Tppp3-CreER*^*T2*^*/Rosa26-stop-tdTomato/Rosa26-stop-rtTA/Col1a1::TetO-β-catenin*). At 1 month of age, the mice were injected with 1 mg of tamoxifen three times, followed by Dox treatment (50 μg/mL). The upper panel shows the mice treated with or without Dox for 1 month. The lower panel showcases two groups: mice treated with Dox for 2 months and those initially treated with Dox for 1 month, followed by a one-month withdrawal of treatment. **b** Masson’s trichrome staining of dermal fibrotic lesions in the paws of Dox-inducible β-catenin-expressing mice (Fig. 3a). Compared with the Dox-off 1 month group (control), the Dox treatment groups showed increased dermal thickness in a time-dependent manner (Dox-on 1 month group; *p* = 0.0123, and Dox-on 2 months group; *p* < 0.0010). Conversely, when compared with the Dox-on 1 month group, the Dox withdrawal group showed halted progression of dermal thickness (Dox-on 1 month + off 1 month group; *p* = 0.8961). The graph shows the relative dermal thickness, with the mean dermal thickness in each sample from the Dox-off 1 month group normalized to 1. The data are presented as the mean ± SD (one-way ANOVA with the Tukey–Kramer multiple comparison test). Biological samples consisted of Dox-off and -on 1 month (*n* = 8) and Dox-on 2 months and -on 1 month + off 1 month (*n* = 6) groups, with dermal thickness defined as the mean measurement across six lesions per sample. **c** Fluorescent immunohistochemistry was performed on dermal fibrotic lesions in the paws of the mice (Fig. 3a). Continuous Dox treatment was associated with the presence of αSMA-expressing fibrotic lesions in the dermis, whereas Dox withdrawal decreased the number of αSMA-expressing myofibroblasts in the dermis. The graph indicates relative myofibroblast counts in the dermis. The data are presented as the mean ± SD (one-way ANOVA with the Tukey–Kramer multiple comparison test). The mean relative myofibroblast count of the Dox-off 1-month group was set to 1. Compared with that in the Dox-off 1 month group, the relative number of myofibroblasts increased in the Dox-on 1 month group significantly (*p* = 0.0216). However, this increase was not statistically significant in the Dox-on 2 months group (*p* = 0.1803). Dox withdrawal (Dox-on 1 month + off 1 month group) decreased the relative number of myofibroblasts, albeit not significantly (*p* = 0.1262). Biological samples encompassed both Dox-off and -on 1 month (*n* = 8) and Dox-on 2 months and -on 1 month + off 1 month (*n* = 6) groups. Two different lesions were analysed per sample. Myofibroblasts were defined as αSMA-positive/CD31-negative cells. **d** A schematic representation of the cell isolation protocol of dermal fibrotic tissues in the forepaws of *Tppp3-CreER*^*T2*^*/Rosa26-stop-rtTA/Col1a1::TetO-β-catenin* mice. Fibrotic cells were cultured with Dox-containing media (0.2 µg/mL) and expanded for 2–3 weeks. **e** RT-qPCR results show the expression of the β-catenin target gene (*Axin2*), myofibroblast markers (*Acta2*, *Tagln*, and *Myh11*), and fibrotic collagens (*Col1a1* and *Col3a1*). Isolated dermal fibrotic cells (Fig. 3d) were split into two groups: one with continued Dox treatment (Dox-on group) and one with Dox withdrawal (Dox-off group) for 24 h. The expression level of the Dox-off group in each sample was set to 1. The mean ± SD of seven independent biological samples (three technical replicates per sample) are shown (two-tailed paired *t* test). **f** Ki67-positive cell detection using immunocytochemical staining of dermal fibrotic cells (Fig. 3d). Ki67 (green) and αSMA (red). Upper: Dox-off; lower: Dox-on. **g** The Ki67- and αSMA-positive cell ratio of Dox-off and Dox-on groups in Fig. 3f. The mean ± SD values of two independent experiments with three independent biological samples are shown. The Dox-on group showed a significant decrease in the Ki67-positive cell ratio compared with the Dox-off group (Mann–Whitney U test; *p* = 0.0411). The αSMA-positive cell ratio showed no significant difference between the Dox-off and Dox-on groups (two-tailed Mann–Whitney U test; *p* = 0.6991). Scale bars represent 50 µm (**b**, **c**) and 100 µm (**f**).

To examine whether progression of dermal fibrosis depends on Wnt/β-catenin signalling, we created doxycycline (Dox)-inducible β-catenin-expressing mice (*Tppp3-CreER*^*T2*^*/Rosa26-stop-tdTomato/Rosa26-stop-rtTA/Col1a1::TetO-β-catenin*) (Fig. 3a; Supplementary Fig. 3a). Similar to that in *Tppp3-CreER*^*T2*^*/Rosa26-stop-tdTomato/β-catenin ex3*^*flox*^ mice, tamoxifen and Dox administration induced dermal fibrosis of the paw in a month (Fig. 3b, c). Dox administration for an additional month elicited a notable increase in dermal thickness while concurrently exerting no remarkable impact on αSMA-expressing myofibroblast counts (Fig. 3b, c). Conversely, Dox withdrawal resulted in the cessation of fibrosis progression (Fig. 3b, c). These findings underscore the pivotal role of Wnt/β-catenin signalling activity in both the development and progression of fibrosis.

Next, we investigated whether myofibroblast phenotype and activity are directly regulated by Wnt/β-catenin signalling. Murine dermal fibrosis tissues derived from Dox-inducible β-catenin-expressing mice were harvested, cultured in vitro, and treated with or without Dox (Fig. 3d, Supplementary Fig. 3b–d). Gene expression analyses using real-time PCR revealed that Dox treatment upregulated the expression of *Axin2*, a target gene of β-catenin (Fig. 3e). In addition, Dox treatment significantly upregulated the expression of myofibroblast markers (*Acta2, Tagln*, and *Myh11*)^18^, type I collagen (*Col1al*), and type III collagen (*Col3a1*) (Fig. 3e). Conversely, in αSMA-expressing myofibroblasts derived from dermal fibrous tissues, Ki67 immunostaining showed that Wnt/β-catenin signalling activation slightly reduced cell proliferation (Fig. 3f, g), which is inconsistent with previous findings^19^. Collectively, these findings indicate that Wnt/β-catenin signalling positively regulates myofibroblast phenotype and collagen expression in murine dermal fibrosis of the paw.

### Wnt/β-catenin signalling inhibition in human Dupuytren’s contracture-derived cells does not decrease the expression of myofibroblast markers and collagens in vitro

Based on the results of the murine dermal fibrosis model, Wnt/β-catenin signalling could serve as a therapeutic target for human Dupuytren’s contractures. Therefore, we investigated the effect of small-molecule inhibitors of Wnt/β-catenin signalling on Dupuytren’s contracture-derived cells in vitro. We used PNU-74654, which inhibits the interaction between β-catenin and T cell factor (TCF), and MSAB, which degrades β-catenin directly^20^. Neither inhibitor decreased the expression levels of myofibroblast markers (*ACTA2, TAGLN*, and *MYH11*), nor did they impact the expression of collagens (*COL1A1* and *COL3A1*) (Supplementary Fig. 4a, b). Another inhibitor of Wnt/β-catenin signalling, XAV939, did not degrade β-catenin in human Dupuytren’s contracture-derived cells (Supplementary Fig. 4c). In contrast to mouse data (Fig. 3e), activation of Wnt/β-catenin signalling via Wnt3A administration did not upregulate the expression of myofibroblast markers and collagens (Supplementary Fig. 4d). These findings indicate that the modulation of Wnt signalling may not directly alter the myofibroblast phenotype and collagen expression in human Dupuytren’s contracture-derived cells.

### Wnt/β-catenin signalling activation in *Tppp3*-positive cells causes TGF-β-mediated fibrotic niche formation through macrophage infiltration

**Fig. 4:**
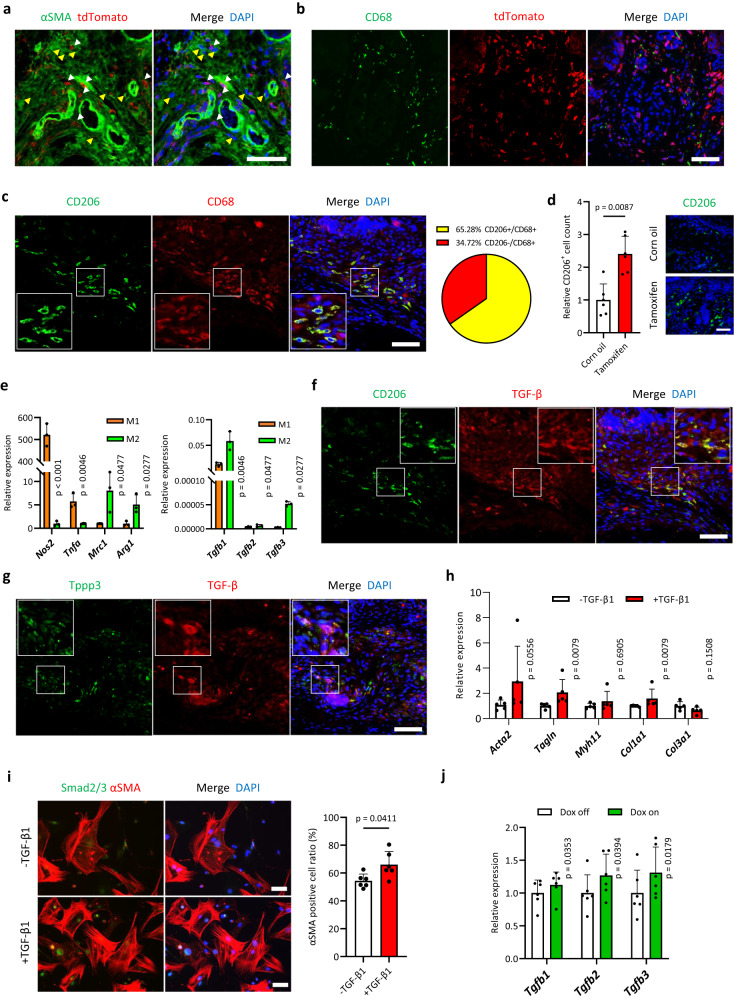
Wnt signalling activation in *Tppp3*-expressing cells leads to the formation of a TGF-β-expressing niche through macrophage infiltration. **a** Fluorescent immunohistochemistry of a dermal fibrotic region in the paw of *Tppp3-CreER*^*T2*^*/Rosa26-stop-tdTomato/Rosa26-stop-rtTA/Col1a1::TetO-β-catenin* mice (Dox-inducible β-catenin mice). tdTomato-positive (white arrowheads) and -negative (yellow arrowheads) αSMA-expressing myofibroblasts were observed. **b** Fluorescent immunohistochemistry of the dermal fibrotic region in the paws of the same Dox-inducible β-catenin mice (Fig. 4a). CD68-expressing macrophages infiltrated around tdTomato-positive cells (β-catenin-expressing *Tppp3*-positive cells). **c** Fluorescent immunohistochemistry showing the macrophage subtypes infiltrating the fibrotic region in *Tppp3-CreER*^*T2*^*/β-catenin ex3*^*flox*^ mice. CD68 (red); pan-macrophage marker, CD206 (green); M2 macrophage marker. An average of 65% of the macrophages comprised M2 macrophages (yellow). The white square area is enlarged in the lower left panel. **d** Quantification of the number of CD206-expressing cells in the dermis of *Tppp3-CreER*^*T2*^*/β-catenin ex3*^*flox*^ mice treated with corn oil or tamoxifen. Activation of β-catenin in *Tppp3*-positive cells significantly increased M2 macrophage infiltration in the dermis (two-tailed Mann–Whitney U test; *p* = 0.0087). The number of CD206-positive cells was counted in two random fields per sample. The means ± SD of three independent biological samples are shown. **e** Gene expression analyses using real-time PCR of M1 and M2 macrophages. RAW267.4 cells were differentiated into M1 macrophages with lipopolysaccharide (60 ng/mL) and M2 macrophages with interleukin-4 (40 ng/mL) for 30 h. *Nos2* and *Tnfa* are M1 macrophage markers, whereas *Mrc1* (CD206) and *Arg1* are M2 macrophage markers. M2 macrophages expressed significantly higher *Tgfb1-3* than M1 macrophages (*p* = 0.0046, 0.0477, and 0.0277, respectively). The mean ± SD values of three independent experiments (three technical replicates per experiment) are shown (two-tailed Mann–Whitney U test). **f** Fluorescent immunohistochemistry of the same region in Fig. 4c shows the TGF-β (red)-expressing niche with M2 macrophage (CD206; green) infiltration. Both M2 macrophages and their surrounding cells expressed TGF-β. The white square area is enlarged in the upper right panel. **g** Fluorescent immunohistochemistry of the fibrotic region in *Tppp3-CreER*^*T2*^*/β-catenin ex3*^*flox*^ mice. A part of Tppp3-expressing stromal cells (green) also expressed TGF-β (red). The white square area is enlarged in the upper left panel. **h** Gene expression analyses using real-time PCR of TGF-β1 (10 ng/mL)-treated murine dermal fibrotic cells derived from *Tppp3-CreER*^*T2*^*/β-catenin ex3*^*flox*^ mice in vitro. The mean ± SD values of five independent experiments (three technical replicates per experiment) are shown (two-tailed Mann–Whitney U test). **i** αSMA-positive cell detection by immunocytochemical staining of murine dermal fibrotic cells derived from *Tppp3-CreER*^*T2*^*/β-catenin ex3*^*flox*^ mice. Smad2/3 (green) and αSMA (red). Upper: control (-TGF-β1) group; lower: 10 ng/mL of TGF-β1 treatment (+TGF-β1) group. The αSMA-positive cell ratio was significantly higher in the TGF-β1 treatment group than that in the control group. The mean ± SD values of two fields for three independent experiments are shown (two-tailed Mann–Whitney U test; *p* = 0.0411). **j** *Tgfb1*, *Tgfb2*, and *Tgfb3* expression in murine dermal fibrotic cells derived from Dox-inducible β-catenin mice (Fig. 3d) was analysed using RT-qPCR. Significant differences were observed between the Dox-off and Dox-on groups (*p* = 0.0353, 0.0394, and 0.0179, respectively). The mean ± SD values of six independent biological samples (three technical replicates per sample) are shown (two-tailed paired *t* test). Scale bars represent 50 µm (**a**, **b**, **c**, **f**, **g**) and 100 µm (**i**).

Although Wnt/β-catenin signalling caused dermal fibrosis of the paw and regulated the myofibroblast phenotype in mice, it could not serve as a therapeutic target for human Dupuytren’s contractures. Detailed histological analyses of fibrotic regions in mice demonstrated that αSMA-positive myofibroblasts consisted of both *Tppp3*-lineage cells (β-catenin-induced cells) and non-*Tppp3*-lineage cells (β-catenin-non-induced cells), indicating that a non-cell-autonomous mechanism may also participate in fibrosis development induced by Wnt/β-catenin signalling activation (Fig. 4a). Recent studies have shown that interactions between stromal cells and immune cells are involved in fibrotic disease, including Dupuytren’s contractures^12,14,21–23^. Consistent with previous findings, many macrophages expressing CD68 (a pan-macrophage marker) were present in fibrotic regions whose formation was induced by Wnt/β-catenin signalling activation (Fig. 4b). Approximately 65% of the macrophages were alternatively activated macrophages (M2) expressing CD206, and their number was increased by Wnt/β-catenin signalling activation (Fig. 4c, d). In vitro analyses showed that M2 macrophages showed higher expression of *Tgfb1, Tgfb2*, and *Tgfb3* than classically activated (M1) macrophages, with *Tgfb1* as the predominant isoform (Fig. 4e). Fibrotic regions with CD206-expressing M2 macrophage infiltration expressed TGF-β (Fig. 4f). Moreover, both M2 macrophages and Tppp3-expressing stromal cells around macrophages expressed TGF-β (Fig. 4f, g). In human Dupuytren’s contracture tissues, macrophages were infiltrated in fibrotic lesions, and TGF-β-expressing niche with TPPP3-expressing stromal cells and CD206-expressing M2 macrophages was observed (Supplementary Fig. 5a–c). In particular, their niche was predominantly localized around CD31-expressing perivascular regions (Supplementary Fig. 5d).

Treatment of murine dermal myofibroblasts and human Dupuytren’s contracture-derived cells with TGF-β1, as a known major inducer of fibrosis, significantly upregulated the expression of myofibroblast markers and collagens and induced αSMA-positive myofibroblast differentiation (Fig. 4h, i, Supplementary Fig. 6a–c). In vitro experiments showed that Wnt/β-catenin signalling activation in murine dermal myofibroblasts upregulated the expression of *Tgfb1*, *Tgfb2*, and *Tgfb3* (Fig. 4j). In contrast, its activation in human Dupuytren’s contracture-derived cells did not upregulate the expression of these genes (Supplementary Fig. 6d). These findings suggest that TGF-β-expressing niche formation may be closely associated with stromal cell–macrophage interaction rather than a cell-autonomous mechanism that occurs via the activation of Wnt/β-catenin signalling. Moreover, TGF-β1 induced the nuclear translocation of β-catenin (Supplementary Fig. 6e), indicating that a positive interaction between Wnt/β-catenin and TGF-β signalling may be related to dermal fibrosis development and progression.

### Depletion of TGF-β signalling partially mitigates Wnt/β-catenin signalling-induced fibrosis in mice

**Fig. 5:**
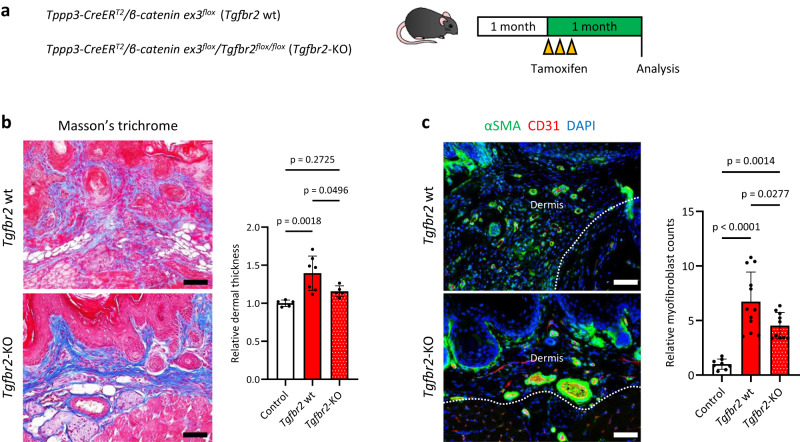
Depletion of TGF-β signalling partially mitigates Wnt/β-catenin signalling-induced fibrosis in mice. **a** A schematic of the in vivo experiment on *Tppp3-CreER*^*T2*^*/β-catenin* ex3 ^*flox*^ mice carrying a conditional deletion allele of TGF-β signalling (*Tgfbr2*^*flox/flox*^). At 1 month of age, the mice were injected with 1 mg of tamoxifen three times and analysed 1 month after injections. **b** Masson’s trichrome staining of the palmar dermis of the paws. The upper panel shows a tamoxifen-treated *Tppp3-CreER*^*T2*^*/β-catenin ex3*^*flox*^ mouse (*Tgfbr2* wt), and the lower panel shows a tamoxifen-treated *Tppp3-CreER*^*T2*^*/β-catenin ex3* ^*flox*^*/Tgfbr2*^*flox/flox*^ mouse (*Tgfbr2*-KO). Corn oil-treated *Tppp3-CreER*^*T2*^*/β-catenin ex3*^*flox*^ mice served as the control group. Relative dermal thickness in each group was analysed using a one-way ANOVA with the Tukey–Kramer multiple comparison test. The mean thickness of the control group was set to 1. Compared with that in *Tgfbr2* wt mice, dermal thickness was decreased significantly in *Tgfbr2*-KO mice (*p* = 0.0496). Biological samples encompassed control (*n* = 5), *Tgfbr2* wt (*n* = 7), and *Tgfbr2*-KO (*n* = 5) groups. Dermal thickness was defined as the mean of six lesions measured per sample. **c** αSMA and CD31 immunohistochemistry of the paws. The upper panel shows a *Tgfbr2* wt mouse, and the right panel shows a *Tgfbr2*-KO mouse. Myofibroblasts were identified as αSMA (green)-positive/CD31 (red)-negative cells. Relative myofibroblast counts in the dermis were analysed using a one-way ANOVA with the Tukey–Kramer multiple comparison test. The mean relative myofibroblast count of the control group was set to 1. Compared with that in *Tgfbr2* wt mice, the relative number of myofibroblasts decreased significantly in *Tgfbr2*-KO mice (*p* = 0.0277). However, the relative number of myofibroblasts in *Tgfbr2*-KO mice was notably higher than that in control mice, indicating that the depletion of TGF-β signalling did not entirely rescue Wnt/β-catenin signalling-induced fibrosis. Biological samples included control (*n* = 4), *Tgfbr2* wt (*n* = 6), and *Tgfbr2*-KO (*n* = 5) groups. Two different lesions were analysed per sample. Scale bars represent 50 µm (**b**, **c**). The data are presented as the mean ± SD (**b**, **c**).

To further examine whether Wnt/β-catenin signalling activation induced fibrosis via TGF-β-expressing niche formation, we generated Wnt/β-catenin and TGF-β signalling-double conditional mice (*Tppp3-CreER*^*T2*^*/β-catenin ex3*^*flox*^*/Tgfbr2*^*flox/flox*^: *Tgfbr2*-knockout [KO] mice) and compared their phenotypes with those of *Tppp3-CreER*^*T2*^*/β-catenin ex3*^*flox*^ mice (*Tgfbr2* wt mice) at 1 month after tamoxifen administration (Fig. 5a, Supplementary Fig. 7a–c). Compared with those in *Tgfbr2* wt mice, the dermal fibrotic layer thickness and the number of αSMA-expressing dermal myofibroblasts in *Tgfbr2*-KO mice decreased significantly (Fig. 5b, c). Meanwhile, *Tgfbr2*-KO mice continued to develop dermal fibrosis comprising myofibroblasts (Fig. 5c), indicating that Wnt/β-catenin signalling activation also initiated an independent fibrotic program not involved in TGF-β signalling. Together, these results suggest that Wnt/β-catenin signalling activation induces fibrosis via both a cell-autonomous fibrotic program and a non-cell-autonomous fibrotic program mediated by TGF-β-expressing niche formation in mice.

### *Tppp3*-positive cells secrete Cxcl14 via Wnt/β-catenin signalling activation

**Fig. 6:**
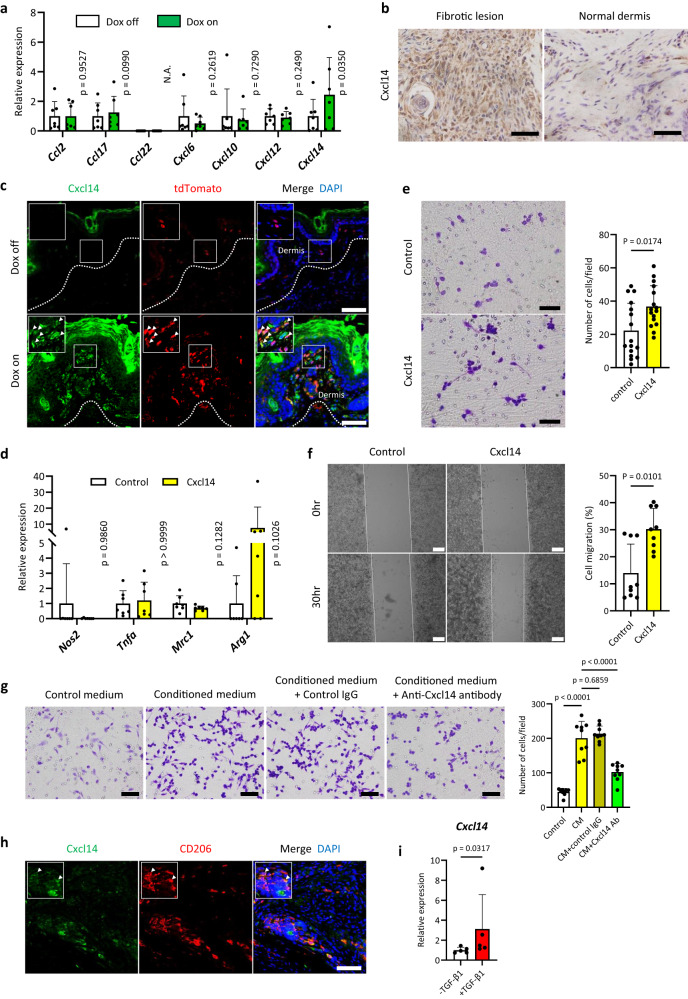
Dermal fibrotic cells express *Cxcl14* via Wnt signalling activation. **a** The expression (RT-qPCR) of M2 macrophage-associated chemokines. Isolated dermal fibrotic cells from the paws of doxycycline (Dox)-inducible β-catenin mice (Fig. 3d) were divided into Dox-on and Dox-off groups. The expression level in the Dox-off group was set to 1. The mean ± SD of seven biological samples (three technical replicates per sample) are shown (two-tailed paired *t* test). **b** Immunohistochemistry of Cxcl14 in dermal fibrotic lesions of the paw in *Tppp3-CreER*^*T2*^*/β-catenin ex3*^*flox*^ (left) and control mice (*Tppp3-CreER*^*T2*^*/Rosa26-stop-tdTomato*) (right). Samples were obtained 1 month after three tamoxifen injections of 1 mg. Marked Cxcl14 expression was detected in dermal fibrotic lesions (left). **c** Fluorescent immunohistochemistry was employed to visualize the distribution of Cxcl14 (green) and tdTomato (red) in the context of dermal fibrosis in the paw of *Tppp3-CreER*^*T2*^*/Rosa26-stop-tdTomato/Rosa26-stop-rtTA/Col1a1::TetO-β-catenin* mice (upper panel: Dox-off mice; lower panel: Dox-on mice). Dox-treated tdTomato-positive cells (β-catenin-expressing *Tppp3*-lineage cells) in the dermis co-expressed Cxcl14 (indicated by white arrowheads). The white square area is enlarged in the upper right panel. **d** Gene expression analysis by RT-qPCR of recombinant Cxcl14-treated (10 nM) murine macrophages (RAW264.7). Cxcl14 treatment did not induce M2 macrophage differentiation. The mean ± SD values of seven independent experiments (three technical replicates per sample) are shown (two-tailed Mann–Whitney U test). **e** Chemotaxis assay of recombinant Cxcl14-treated (10 nM) macrophages. Compared to the control group (upper panel), Cxcl14 treatment (lower panel) showed significantly increased cell infiltration (*p* = 0.0174). The mean ± SD values of four independent experiments (three different lesions per experiment) are shown (two-tailed Mann–Whitney U test). **f** Migration assay of recombinant Cxcl14-treated (10 nM) macrophages. Compared to the control (left), the Cxcl14 treatment (right) showed significantly increased cell migration after 30 h (*p* = 0.0101). The mean ± SD of three independent experiments (three different lesions per experiment) are shown (two-tailed Mann–Whitney U test). **g** Chemotaxis assay of control IgG and anti-Cxcl14 antibody-treated macrophages. Compared to the control medium, the conditioned medium significantly increased cell infiltration (*p* < 0.0001). Compared to the conditioned medium alone, control IgG treatment did not yield a statistically significant reduction in cell infiltration (*p* = 0.6859). Conversely, anti-Cxcl14 antibody treatment resulted in a significant decrease in cell infiltration (*p* < 0.0001). The mean ± SD values of three independent experiments (three different lesions per experiment) are shown (one-way ANOVA with Tukey–Kramer multiple comparison test). CM; conditioned medium. **h** Fluorescent immunohistochemistry of Cxcl14 (green) and CD206 (red) in the context of dermal fibrosis in the paw of *Tppp3-CreER*^*T2*^*/β-catenin ex3*^*flox*^ mice (1 month after tamoxifen injection). CD206-expressing M2 macrophages were polarized around Cxcl14-expressing cells. Some M2 macrophages co-expressed Cxcl14 (indicated by white arrowheads). The white square area is enlarged in the upper right panel. **i** *Cxcl14* expression (RT-qPCR) in recombinant TGF-β1 (10 ng/mL)-treated murine dermal fibrotic cells derived from *Tppp3-CreER*^*T2*^*/β-catenin ex3*^*flox*^ mice. TGF-β1 treatment significantly upregulated *CXCL14* expression (*p* = 0.0317). The mean ± SD of six independent biological samples (three technical replicates per sample) are shown (two-tailed Mann–Whitney U test). Scale bars represent 20 µm (**b**), 50 µm (**c**, **f**, **g**), or 200 µm (**e**).

We sought to investigate the mechanism by which Wnt/β-catenin signalling activation in *Tppp3*-positive cells leads to macrophage infiltration. Macrophage differentiation and recruitment are induced by chemotactic factors, including cytokines and chemokines^23^. In our fibrosis model, infiltrating macrophages were predominantly of the M2 subtype; therefore, we examined the expression of chemokines associated with M2 macrophage differentiation and recruitment. *Cxcl14* expression was significantly upregulated in Dox-treated murine dermal fibrotic cells in vitro (Fig. 6a), and Cxcl14 expression was increased in murine dermal fibrotic regions induced by Wnt/β-catenin signalling activation in vivo (Fig. 6b, c). Similarly, *CXCL14* expression was detected in human Dupuytren’s contracture samples (Supplementary Fig. 8a, b). CXCL14 expression increased in β-catenin-expressing cells in the nodule compared with that in non-diseased cells in the palmar fascia (Supplementary Fig. 8c, d), although it was detected in both αSMA-positive and -negative stromal cells in the nodule (Supplementary Fig. 8e). In addition, Wnt3A treatment upregulated *CXCL14* expression in human Dupuytren’s contracture-derived cells in vitro (Supplementary Fig. 8f, g).

Although recombinant Cxcl14 treatment did not induce differentiation of macrophages, it promoted the migration and chemotaxis of macrophages in vitro (Fig. 6d–f). Additionally, blockade of Cxcl14 by the anti-Cxcl14 antibody decreased the chemotaxis of macrophages (Fig. 6g). Consistent with the results in vitro, CD206-expressing M2 macrophages were polarized around Cxcl14/CXCL14-expressing stromal cells in mouse fibrotic lesions and human Dupuytren’s contracture tissues (Fig. 6h, Supplementary Fig. 8h). Moreover, some M2 macrophages co-expressed Cxcl14/CXCL14 (Fig. 6h, Supplementary Fig. 8h). Interestingly, TGF-β1 also markedly upregulated Cxcl14/CXCL14 expression in both murine dermal fibrotic cells and human Dupuytren’s contracture-derived cells, which was supported by the nuclear translocation of β-catenin via TGF-β1-mediated Wnt/β-catenin signalling activation (Fig. 6i, Supplementary Fig. 6e and 8i). These data suggest that Wnt/β-catenin signalling activation induces the expression of Cxcl14/CXCL14, which is involved in macrophage recruitment in both mice and humans.

### Anti-Cxcl14 neutralizing antibody treatment partially suppresses dermal fibrosis by reducing macrophage infiltration in mice

**Fig. 7:**
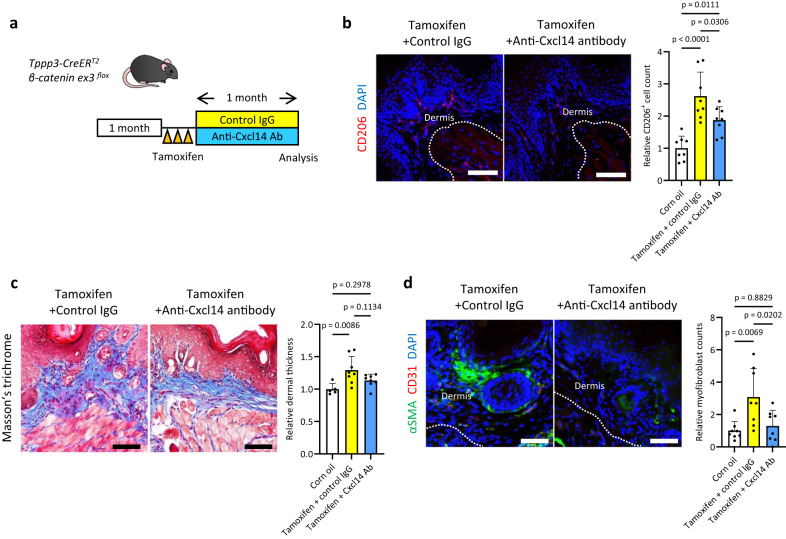
Neutralization of Cxcl14 partially suppresses Wnt/β-catenin-induced dermal fibrosis by inhibiting M2 macrophage infiltration in mice. **a** A schematic of the experimental protocol of anti-Cxcl14 neutralizing antibody treatment for *Tppp3-CreER*^*T2*^*/β-catenin ex3*^*flox*^ mice. Mice were treated with 15 μg of an anti-Cxcl14 neutralizing antibody or control IgG twice a week for 1 month. **b** Fluorescent immunohistochemistry of CD206 (red) in the paws. The left panel shows control IgG treatment, and the right shows anti-Cxcl14 neutralizing antibody treatment after tamoxifen injections. Dotted lines indicate the border of the dermis. The relative CD206-positive cell count in each group was analysed (one-way ANOVA with the Tukey multiple comparison test). Biological samples comprised corn oil (*n* = 4), tamoxifen + control IgG (*n* = 4), and tamoxifen + Cxcl14 antibody (*n* = 4) groups. Two different lesions were analysed per sample. **c** Masson’s trichrome staining of the dermal fibrotic lesion of the paws. The left panel shows control IgG treatment, and the right shows anti-Cxcl14 neutralizing antibody treatment. Relative dermal thickness in each group was analysed (one-way ANOVA with the Tukey–Kramer multiple comparison test). Compared to the control treatment (corn oil), control IgG treatment did not exhibit any protective effect against Wnt/β-catenin-induced fibrosis (*p* = 0.0086), whereas anti-Cxcl14 neutralizing antibody treatment demonstrated a discernible protective effect in this context (*p* = 0.2978). The mean thickness of the control group was set to 1. Biological samples comprised corn oil (*n* = 5), tamoxifen + control IgG (*n* = 8), and tamoxifen + Cxcl14 antibody (*n* = 8) groups. Dermal thickness was defined as the mean of four lesions measured per sample. **d** Fluorescent immunohistochemistry of αSMA (green) and CD31 (red) in the dermis of the paws. Left: control IgG treatment after tamoxifen injections; lower: anti-Cxcl14 neutralizing antibody treatment after tamoxifen injections. Myofibroblasts were defined as αSMA-positive/CD31-negative cells. Relative myofibroblast counts in the dermis were analysed (one-way ANOVA with the Tukey multiple comparison test). Compared with the control IgG treatment, anti-Cxcl14 antibody treatment significantly decreased the number of myofibroblasts in the dermis (*p* = 0.0202). Biological samples included corn oil (*n* = 4), tamoxifen + control IgG (*n* = 4), and tamoxifen + Cxcl14 antibody (*n* = 4) groups. Two different lesions were analysed per sample. Scale bars represent 50 µm (**b**, **c**, **d**). The data are presented as the mean ± SD (**b**, **c**, **d**).

Finally, we investigated whether blockade of Cxcl14 in Wnt/β-catenin signalling in a fibrotic mouse model can suppress dermal fibrosis by reducing macrophage infiltration. *Tppp3-CreER*^*T2*^*/β-catenin ex3*^*flox*^ mice administered tamoxifen were treated with an anti-Cxcl14 neutralizing antibody or control IgG for 1 month, and dermal fibrotic lesions of the paws were investigated (Fig. 7a). As expected, anti-Cxcl14 neutralizing antibody treatment decreased CD206-expressing M2 macrophage infiltration into the dermis (Fig. 7b). Compared with the corn oil treatment, the control IgG treatment showed significant thickening of the dermis on the paw, whereas the anti-Cxcl14 neutralizing antibody treatment partially suppressed Wnt/β-catenin-induced dermal thickening of the paw (Fig. 7c). We found fewer αSMA-expressing myofibroblasts in mice treated with the anti-Cxcl14 neutralizing antibody than in those treated with the control IgG (Fig. 7d). These findings imply that neutralization of Cxcl14 partially suppresses Wnt/β-catenin signalling-induced fibrosis by inhibiting M2 macrophage infiltration.

## Discussion

**Fig. 8:**
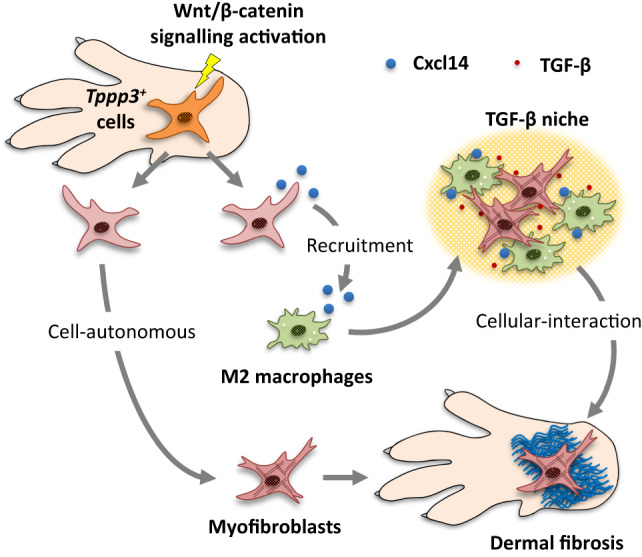
Schematic representation of the cell- and non-cell-autonomous fibrotic program induced by Wnt/β-catenin signalling activation in *Tppp3*-positive cells.

The cellular origin of Dupuytren’s contracture and the significance of Wnt signalling activation in this disease remain elusive. In this study, we found that activation of Wnt/β-catenin signalling in *Tppp3*-positive cells in the dermis of the paw caused dermal fibrosis resembling human Dupuytren’s contractures. Moreover, the development and progression of the disease were shown to involve the following: (1) a cell-autonomous fibrotic program induced by Wnt/β-catenin signalling activation in *Tppp3*-positive cells; and (2) a non-cell-autonomous fibrotic program mediated by TGF-β, which was released based on the interaction between *Tppp3*-positive stromal cells and macrophages via the chemokine *Cxcl14*, induced by Wnt/β-catenin signalling activation in *Tppp3*-positive cells (Fig. 8).

Wnt/β-catenin signalling is an important pathway in organ development and cellular differentiation^24^. Dysregulation of Wnt/β-catenin is involved in fibrosis in various organs and tissues in mice, rats, and humans^19,25,26^. Wnt/β-catenin signalling activation is associated with cell proliferation and migration and increased production of collagen and matrix^19,27,28^. Furthermore, it directly induces myofibroblast differentiation^19^. Conditional knockout of β-catenin and administration of Wnt/β-catenin signalling inhibitors (ICG-001, PRI-724) ameliorated bleomycin-induced murine dermal and lung fibrosis and chronic hepatitis C virus-induced liver fibrosis in vivo^29–32^. In contrast, some studies have reported that the expression of αSMA, a typical myofibroblast marker, was not directly induced by activation of Wnt/β-catenin signalling via Wnt3A^28,33,34^. Our study showed that the myofibroblast phenotype was directly induced by Wnt/β-catenin signalling in mice but not in humans. Verjee et al.^13^ demonstrated that tumour necrosis factor (TNF) treatment for palmar dermal fibroblasts derived from patients with Dupuytren’s disease led to activation of Wnt/β-catenin signalling through GSK-3β phosphorylation and inhibition, resulting in upregulated COL1 and α-SMA expression. Meanwhile, activation of Wnt/β-catenin signalling by a GSK-3β inhibitor (SB-216763) did not induce COL1 and α-SMA expression. These findings suggest that the role of Wnt/β-catenin signalling in fibrosis might differ across cell types, biological species, and fibrosis models. Moreover, in addition to Wnt/β-catenin-mediated cell-autonomous myofibroblast differentiation, alternative mechanisms may exist for fibrosis development and progression.

TGF-β is closely associated with fibrosis and is a representative fibrosis inducer^35^. All three TGF-β isoforms induce a fibrotic response, although the role of TGF-β3 as a fibrosis inducer or repressor remains controversial^36^. Interestingly, TGF-β stimulated Wnt signalling by inducing the nuclear accumulation of β-catenin in rat pulmonary alveolar cells and human fibroblasts^37,38^ and induced αSMA expression by interacting with Smad3, CBP, and β-catenin^38^. Therefore, β-catenin is essential for TGF-β-mediated fibrosis. Similarly, we showed that TGF-β1 treatment induced nuclear translocation of β-catenin in human Dupuytren’s contracture-derived cells. Myofibroblast differentiation via Wnt3A was observed in the presence of TGF-β^34^. Our in vivo analysis using *Tppp3-CreER*^*T2*^*/β-catenin ex3* ^*flox*^*/Tgfbr2*^*flox/flox*^ (*Tgfbr2*-KO) mice demonstrated that TGF-β signalling was associated with Wnt/β-catenin-induced fibrosis. Together, these findings suggest that Wnt/β-catenin signalling and TGF-β signalling are intimately related to fibrosis and could be potential therapeutic targets for fibrotic disease. However, in this study, Wnt signal inhibitors PNU-74654, MSAB, and XAV939 did not reduce the expression of myofibroblast markers (including αSMA) and collagen in human Dupuytren’s contractures. Moreover, using TGF-β inhibitors for molecular therapy is challenging owing to the crucial role of TGF-β in the maintenance of cell homeostasis^22^. Thus, targeting these signals directly in the treatment of Dupuytren’s contractures may prove difficult.

Recently, the importance of stromal cell–immune cell interactions has been recognized in fibrotic disease development and progression^23,39^. Chronic inflammation by immune cells (particularly macrophages) is involved in fibrosis development in several organs and tissues, including pulmonary fibrosis, skin fibrosis, and liver fibrosis^23,39–41^. Dupuytren’s contractures share this pathological process of the development and progression of fibrosis^12–14^. Izadi et al.^12^ reported reciprocal activating pathways through stromal cells-expressed IL-33 and TNF-expressing M2 macrophages and mast cells. Furthermore, they demonstrated that anti-IL-33 and TNF receptor II antibody treatment downregulated the expression of fibrotic genes and reduced cellular contractility of Dupuytren’s myofibroblasts. Akbar et al.^14^ showed that mast cell-expressed IL-13 activated myofibroblasts through STAT signalling and that tofacitinib, an inhibitor of JAK1 and JAK3 related to the phosphorylation of STAT1 and STAT6, reduced proliferation and fibrotic gene expression in Dupuytren’s myofibroblasts. Thus, it is expected that the stromal cell–immune cell interaction can serve as a novel therapeutic target for Dupuytren’s disease.

In this study, we found that Wnt/β-catenin signalling activation in *Tppp3*-positive cells recruited macrophages and formed a TGF-β-expressing niche via Cxcl14. Macrophages are classified into two subtypes: classically activated macrophages (M1) and alternatively activated macrophages (M2). TGF-β is predominantly expressed in M2 macrophages, and its expression in these macrophages increases when they are in contact with fibroblasts^42^. Macrophage differentiation and recruitment are induced by several kinds of cytokines and chemokines^43–47^. *Cxcl14/CXCL14* is the most recently identified C-X-C chemokine and is highly conserved in mice and humans^48^. Cereijo et al. ^43^ and Wang et al. ^47^ demonstrated that Cxcl14, secreted by brown adipocytes and pericytes, recruits macrophages and differentiates them into the M2 subtype, indicating that it could be an inducer of M2 macrophage polarization. Here, we demonstrated that *Cxcl14/CXCL14* expression was upregulated via Wnt/β-catenin activation in murine dermal *Tppp3*-positive cells and human Dupuytren’s contracture-derived cells. In addition, TGF-β1 upregulated *Cxcl14/CXCL14* expression in murine dermal fibrotic cells and human Dupuytren’s contracture-derived cells. Thus, a TGF-β-expressing niche, induced by Wnt/β-catenin signalling activation, may be stabilized via a positive-feedback interaction between stromal cells and macrophages through *Cxcl14/CXCL14*.

*Cxcl14* is involved in the progression of murine liver fibrosis^49^. However, no study thus far has analysed its function in disease development and progression. Notably, knockdown of *Cxcl14* expression and neutralization of Cxcl14 via antibodies ablated macrophage migration caused by the senescence-associated secretory phenotype both in vitro and in vivo, respectively^50^. Consistent with this report, we revealed that the neutralization of Cxcl14 in *Tppp3-CreER*^*T2*^/*β-catenin ex3*^*flox*^ mice reduced M2 macrophage infiltration and mitigated dermal fibrosis in vivo. Moreover, CXCL14 is highly expressed in human idiopathic lung fibrosis^51^, and its expression was observed in Dupuytren’s contractures^52^, suggesting that it may also be associated with macrophage infiltration in human diseases.

This study has some limitations. First, we generated a mouse fibrosis model with β-catenin gene mutations; however, β-catenin gene mutations are absent in human Dupuytren’s contractures^8,53^. This limitation may have been responsible for the differences in myofibroblast differentiation and induction of TGF-β expression in response to Wnt/β-catenin signalling activation between mice and humans in this study. Therefore, to analyze the development and progression of Dupuytren’s contracture smore accurately, it may be necessary to create alternative mouse models, focusing on known SNPs in Dupuytren’s contractures^6,7^.

Second, Cxcl14 inhibition did not completely prevent M2 macrophage infiltration and fibrosis development; therefore, other factors controlling stromal cell–immune cell interaction could be involved in Wnt/β-catenin-induced fibrosis. Furthermore, Wnt/β-catenin signalling-induced fibrosis involves both cell- and non-cell-autonomous fibrosis programs (Figs. 5, 7, and 8). Therefore, it becomes apparent that exclusively targeting Cxcl14 may not yield a marked therapeutic effect in vivo.

Third, we did not elucidate the precise mechanism through which Wnt/β-catenin signalling regulates *Cxcl14/CXCL14* expression in *Tppp3*-positive cells and human Dupuytren’s contracture-derived cells. Specifically, luciferase reporter constructs containing sequences spanning from +1908 to −292 relative to the start codon of murine *Cxcl14* failed to upregulate promoter activity upon Dox-inducible β-catenin expression and TGF-β1 administration (see Supporting Data). These results suggest the possible existence of other regulatory mechanisms influenced by β-catenin and the TCF/LEF complex or suggest indirect regulation mediated by β-catenin-target genes. Moreover, we did not reveal the precise mechanisms by which *Cxcl14* regulates TGF-β expression in M2 macrophages. Additionally, most of its receptors, with the exception of CXCR4 and IGF-1R^54,55^, remain elusive^48^, leaving the exact downstream pathway unclear. Further analyses are required to investigate the clinical application of *Cxcl14*-associated pathway-targeted therapy.

Fourth, Cxcl14 may play a role in the regulation of the central nervous system, specifically in feeding behavior and host defense against infections^56,57^. In addition, *Cxcl14*-deficient mice exhibited lower weight, high blood glucose levels due to insulin resistance, and reduced bacterial clearance compared with wild-type mice, although the mice were viable^43,58^. Therefore, we should validate the risk of side effects due to blocking Cxcl14 and overcome them for clinical application.

Collectively, identifying *Cxcl14*-mediated cellular interaction between stromal cells and macrophages via activation of Wnt/β-catenin signalling and the formation of a TGF-β niche reflects an important process in the development and progression of fibrotic diseases. This mechanism of cellular crosstalk between stromal cells and macrophages could be a promising therapeutic target for fibrosis, including Dupuytren’s contracture.

## Methods

### Human participants

Experiments using clinical samples of human Dupuytren’s contracture were approved by the Ethics Committee of Gifu University (approval number 28-140). Between 2016 and 2023, the patients underwent surgery for Dupuytren’s contracture in our institution and were informed of this research. We obtained written informed consent from 14 patients (13 men and 1 woman) who agreed to this research, and excised samples were collected at the time of the operation. The patients were as follows: DD-1, 72-year-old female; DD-2, 86-year-old male; DD-3, 76-year-old male; DD-4, 76-year-old male; DD-5, 71-year-old male; DD-6, 75-year-old male; DD-7, 51-year-old male; DD-8, 80-year-old male; DD-9, 71-year-old male; DD-10, 86-year-old male; DD-11, 75-year-old male; DD-12, 70-year-old male; DD-13, 79-year-old male; and DD-14, 55-year-old male. All ethical regulations relevant to human research participants were followed.

### Animals

All animal experiments were approved by the Gifu University Animal Experiment Committee (approval number 2019-183, 2021-094, 2021-238), and the care of the animals was implemented following the Animal Research: Reporting of in Vivo Experiments guidelines. *Rosa26-LSL-tdTomato* (Ai9), *Rosa26-M2rtTA*, and *Rosa26-LSL-rtTA-ires-EGFP* mice were purchased from Jackson Laboratory (https://www.jax.org/). *Tppp3-CreER*^*T2*^ mice and doxycycline-inducible constitutive active β-catenin (S33 mutation) mice (*Col1a1::TetO-β-catenin*) were previously established^59,60^. *β-catenin exon3* floxed mice (*β-catenin ex3*^*flox*^)^61,62^ and *Tgfbr2* floxed mice (*Tgfbr2*^*flox*^)^63,64^ were kindly gifted by Makoto M. Taketo and Harold L. Moses, respectively. For the tamoxifen-inducible Cre-recombination experiment, 1-month-old mice were treated with 1 mg of tamoxifen (Sigma–Aldrich) or corn oil as a control (Wako) for three consecutive days. For doxycycline (Dox; Wako)-inducible β-catenin induction, the mice were treated with Dox-containing water at 50 µg/mL for 1–2 months after Cre-recombination. Both male and female mice were randomly used without bias.

### Cell lines

Dermal fibrotic tissues were dissected from the dermal layer of the palmar paws of constitutive active β-catenin-expressing mice (*Tppp3-CreER*^*T2*^*/β-catenin ex3* ^*flox*^) and Dox-inducible β-catenin-expressing mice (*Tppp3-CreER*^*T2*^*/Rosa26-stop-tdTomato/Rosa26-stop-rtTA/Col1a1::TetO-β-catenin*) under a surgical microscope and dissociated with collagenase for 30 min at 37 °C. Next, they were cultured in a 12-well plate (Thermo Fisher Scientific) with Dulbecco’s modified Eagle’s medium (DMEM) (Wako) supplemented with 10% fetal bovine serum (FBS) and 1% penicillin/streptomycin for approximately 2 weeks. Dermal fibrotic cells derived from Dox-inducible β-catenin-expressing mice were supplemented with 0.2 µg/mL Dox.

The RAW264.7 murine macrophage cell line was purchased from American Type Culture Collection (https://www.atcc.org/). They were maintained in DMEM (Wako) supplemented with 10% FBS and 1% penicillin/streptomycin.

Surgically resected human Dupuytren’s contracture tissues were obtained. From these tissues, nodular regions were shredded into 2–3 mm pieces and dissociated with collagenase for 30 min at 37 °C. Next, they were cultured in a 10 cm dish (Thermo Fisher Scientific) with DMEM supplemented with 10% FBS and 1% penicillin/streptomycin for approximately 2 weeks.

### In vitro experiments

Murine dermal fibrotic (1st passage) and human Dupuytren’s (1st or 2nd passage) cells were reseeded in 6-, 12-, or 24-well plates for subsequent analyses. Murine dermal fibrotic cells were treated with (0.2 µg/mL) or without Dox for 24 h (*n* = 7 independent samples) and 10 ng/mL TGF-β1 (Proteintech) for 24 h (*n* = 5 independent samples). Human Dupuytren’s cells were treated with dimethyl sulfoxide, Wnt inhibitors (0.2 μM and 2.0 µM XAV939 [Wako], 1 μM MSAB [Sigma–Aldrich], 25 μM PNU-74654 [Sigma–Aldrich]), 100 ng/mL recombinant Wnt3A (R&D Systems), and 1 ng/mL and 10 ng/mL TGF-β1 (Proteintech) for 24 h (*n* = 4–7 independent samples). Subsequent analyses, including RNA expression profiling, Western blotting, and immunocytochemistry, were performed on these cells.

For M1 and M2 macrophage differentiation, RAW264.7 murine macrophage cells were treated with RPMI-1640 medium (Thermo Fisher Scientific) supplemented with 60 ng/mL lipopolysaccharide (Sigma–Aldrich) and 40 ng/mL interleukin-4 (PeproTech) for 30 h each.

### Migration and chemotaxis assay

The migration and chemotaxis assay were performed as previously described^43,47^. For the migration assay, the macrophages were cultured on 12-well plates. At 100% confluence, they were treated with RPMI-1640 medium (Thermo Fisher Scientific) and supplemented with or without 10 nM recombinant mouse CXCL14/BRAK protein (R&D Systems). The bottom of the plates was scratched with a 200 µL micropipette tip and analysed at 0 h and 30 h after scratching.

For the chemotaxis assay shown in Figs. 6e, 1.5 × 10^5^ (200 µL) macrophage cells were placed into Costar Transwell chambers on 24-well plates (Corning Incorporated, NY, USA). The bottom wells of the plates were filled with serum-free medium, with or without 10 nM recombinant mouse CXCL14/BRAK protein (R&D Systems). Afterward, the plates were incubated at 37 °C for 30 h. In the chemotaxis assay depicted in Fig. 6g, a total of 3.0 × 10^4^ macrophages (200 µL) were seeded into Costar Transwell chambers on 24-well plates. The bottom wells of the plates were filled with 10% FBS containing DMEM (control medium), conditioned medium alone, and conditioned medium with 20 µg/mL of a control IgG (AB-108-C; R&D Systems) or anti-CXCL14 neutralizing antibody (AF866; R&D Systems)^50^. A supernatant culture medium of murine dermal fibrotic cells derived from *Tppp3-CreER*^*T2*^*/β-catenin ex3* ^*flox*^ mice was used as the conditioned medium. The plates were subsequently incubated at 37 °C for 24 h. The transwell chamber containing macrophages was washed with PBS, and the cells were fixed with 2% paraformaldehyde (PFA) for 20 min and stained with 0.2% crystal violet for 5 min at room temperature. Non-migrating cells on the upper side of the membranes were removed by scraping. The membranes were attached to glass slides. Finally, migrating macrophages were counted using a microscope (BX51, Olympus, Tokyo, Japan).

### Real-time quantitative reverse transcription-polymerase chain reaction (RT-PCR)

RNA was extracted using the RNeasy Plus Mini Kit (QIAGEN). Up to 1 µg of RNA was used for reverse transcription into cDNA using the PrimeScript RT reagent kit (TAKARA). Real-time quantitative RT-PCR (RT-qPCR) was performed using Premix Ex Taq™ (Perfect Real Time) (TAKARA). Transcript levels were analysed with three technical replicates and normalized to those of *ACTB*/*Actb*. The primer sequences are listed in Supplementary Table S1.

### Histological analysis and immunohistochemistry

All murine and human tissue samples were fixed with 4% PFA overnight. The samples were decalcified with pH 7.2 EDTA buffer (G-Chelate Mild, GenoStuff) for 2 weeks and embedded in paraffin. The samples were sectioned into 3 μm-thick slices. Haematoxylin and eosin (H&E) staining and Masson’s trichrome staining were performed using standard protocols. Primary antibodies for immunohistochemistry included anti-αSMA [1A4] (Abcam; dilution 1:200), anti-β-catenin [14/Beta-Catenin] (BD Transduction Laboratories; 1:200), anti-TPPP3 [GTX33554] (GeneTex; 1:200), anti-RFP [ARG55744] (Arigo Biolaboratories; 1:250), anti-CD31 [D8V9E] (Cell Signaling Technology; 1:200), anti-CD31 [ab28364] (Abcam; 1:200), anti-CD68 [D4B9C] (Cell Signaling Technology; 1:200), anti-CD68 [E3O7V] (Cell Signaling Technology; 1:200), anti-CD68 [FA-11] (Thermo Fisher Scientific; 1:200), anti-CD206 [E6T5J] (Cell Signaling Technology; 1:200), anti-TGF-β [TB21] (Bio-Rad Laboratories; 1:200), anti-CXCL14 [N3C3] (GeneTex; 1:200), and anti-CXCL14 [MAB730] (R&D systems; 1:150). The samples were incubated with these primary antibodies at 4 °C overnight.

The secondary antibody for 3, 3′-diaminobenzidine staining was Dako EnVision (Dako Japan, Inc., Kyoto, Japan). The samples were incubated at room temperature for 30 min, and the stained cells were analysed using microscopy (BX51, Olympus). For immunofluorescence, the secondary antibodies were conjugated with Alexa Fluor 488 and Alexa Fluor 594 (Thermo Fisher Scientific), followed by incubation with the cells at room temperature for 2 h. Nuclei were counterstained with DAPI (Cell Signaling Technology). The stained cells were analysed using fluorescence microscopy (IX83, Olympus).

### Immunocytochemistry

Cultured cells were washed with PBS and fixed with 2% PFA for 15 min at room temperature. Next, the cells were treated with a blocking reagent containing 1% bovine serum albumin (BSA, Sigma–Aldrich) for 1 h at room temperature. Antibodies used for immunocytochemistry included anti-αSMA [1A4] (Abcam; dilution 1:300), anti-Ki67 [SP6] (Abcam; 1:300), anti-Smad2/3 [D7G7] (Cell Signaling Technology; 1:300), anti-β-catenin [14/Beta-Catenin] (BD Transduction Laboratories; 1:300), anti-TGFBR2 [2D5H7] (Proteintech; 1:200), and anti-CXCL14 [N3C3] (GeneTex; 1:200). The samples were incubated with these primary antibodies at 4 °C overnight. The secondary antibodies were conjugated with Alexa Fluor 488 and Alexa Fluor 594 (Thermo Fisher Scientific), followed by incubation with the cells at room temperature for 2 h. Nuclei were counterstained with DAPI (Cell Signaling Technology). The stained cells were analysed using fluorescence microscopy (IX83, Olympus).

### Western blot analysis

Cultured cells were harvested in 150 µL of RIPA lysis buffer, and the protein concentration was measured. Proteins were denatured with 2× SDS at 95 °C for 5 min, and 20 μg of the denatured protein was loaded onto a 10% SDS-PAGE gel. The separated proteins were transferred to a polyvinylidene fluoride membrane (Amersham Hybond-P polyvinylidene fluoride membrane, GE HealthCare). The membranes were treated with a blocking reagent containing 5% BSA (Sigma–Aldrich) for 1 h at room temperature. Primary antibodies were applied in Can Get Signal Solution 1 (TOYOBO, Osaka, Japan) overnight at 4 °C and secondary antibodies in Can Get Signal Solution 2 (TOYOBO) for 1 h at room temperature. Pierce ECL Plus Western Blotting Substrate (Thermo Fisher Scientific) was used for visualization. Blots were scanned with ImageQuant™ LAS4000 mini4000 (Cytiva) and quantified using ImageJ (https://imagej.nih.gov/ij/index.html).

The primary antibodies used were anti-TPPP3 [GTX33554] (GeneTex; dilution 1:500), anti-αSMA [1A4] (Abcam; 1:2000), anti-β-catenin [14/Beta-Catenin] (BD Transduction Laboratories; 1:2000), anti-LaminB1 [12987-1-AP] (Proteintech; 1:2000), anti-CXCL14 [N3C3] (GeneTex; 1:500), and anti-β-actin [13E5] (Cell Signaling Technology; 1:2000); the secondary antibodies used were HRP-linked anti-rabbit IgG [#7074] and anti-mouse IgG [#7076] antibodies (Cell Signaling Technology; 1:5000).

### Anti-CXCL14 neutralizing antibody treatment in vivo

One-month-old *Tppp3-CreER*^*T2*^/*β-catenin ex3*^*flox*^ mice were treated with 1 mg of tamoxifen for three consecutive days and were administered 15 µg (100 µg/mL; 150 µL) of a mouse monoclonal anti-CXCL14 neutralizing antibody [MAB730] (R&D systems) or 15 µg (100 µg/mL; 150 µL) of mouse monoclonal control IgG [C1.18.4] (Bio X Cell) twice a week for 1 month. All mice were euthanized by cervical dislocation at 2 months of age, and the forepaws were collected.

### Statistics and reproducibility

Data from the RT-qPCR, immunocytochemistry, Masson’s trichrome staining, migration assay, chemotaxis assay, and immunohistochemistry assay are presented as mean ± standard deviation (SD). For the statistical comparison of RT-qPCR data, a parametrical two-tailed paired *t*-test or non-parametrical two-tailed Mann–Whitney U test was used. For the comparison of the Ki67- and αSMA-positive cell ratio of murine dermal fibrotic cells, αSMA-positive cell ratio of human Dupuytren’s contracture-derived cells, migration and chemotaxis assays of RAW264.7 cells, dermal thickness, and number of myofibroblasts and CD206-positive mouse cells in immunocytochemistry, a non-parametrical two-tailed Mann–Whitney U test or one-way analysis of variance (ANOVA) with the Tukey or Tukey–Kramer multiple comparison test was used. Statistical analyses were performed using GraphPad Prism 9.4.0. Differences were considered statistically significant at *p* < 0.05.

### Reporting summary

Further information on research design is available in the Nature Portfolio Reporting Summary linked to this article.

### Supplementary information

Supplementary Information

Description of Additional Supplementary Files

Supporting Data 1

Reporting Summary

## Data Availability

Source data for the graphs are available as Supporting Data file, and uncropped blots are provided in Supplementary Fig. 9. Any remaining data that support the findings of this study are available from the corresponding author upon reasonable request.
