# Supplementary material for: C-X-C domain ligand 14-mediated stromal cell–macrophage interaction as a therapeutic target for hand dermal fibrosis
Source: Commun Biol. 2023 Nov 18;6:1173. doi: 10.1038/s42003-023-05558-8 (PMC10657354; doi:10.1038/s42003-023-05558-8)
Supplement: Supplementary file 1 — Supplementary Information [file 42003_2023_5558_MOESM1_ESM.pdf]

**Supplemental information**

# C-X-C domain ligand 14-mediated stromal cell–macrophage interaction as a therapeutic target for hand dermal fibrosis

Atsushi Goto, Shingo Komura, Koki Kato, Rie Maki, Akihiro Hirakawa, Hiroyuki Tomita,

Akihiro Hirata, Yasuhiro Yamada, Haruhiko Akiyama

**Supplemental Information**

Legends to Supplemental Figures (Supplementary Figs. 1–9)

Legend to Supplemental Table (Supplementary Table 1)

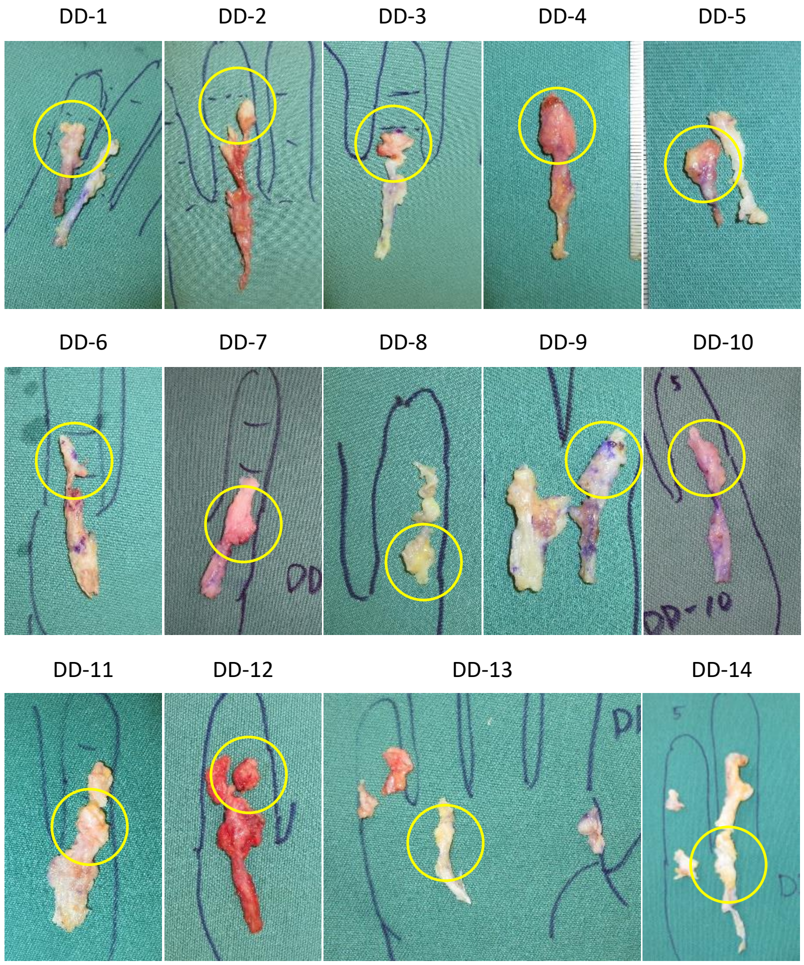


**Supplementary Fig. 1**

**Human Dupuytren's contracture samples analysed in this study.**

Macro-images of 14 surgically excised human Dupuytren's contracture samples. RNA was extracted from nodules (indicated by yellow circles).

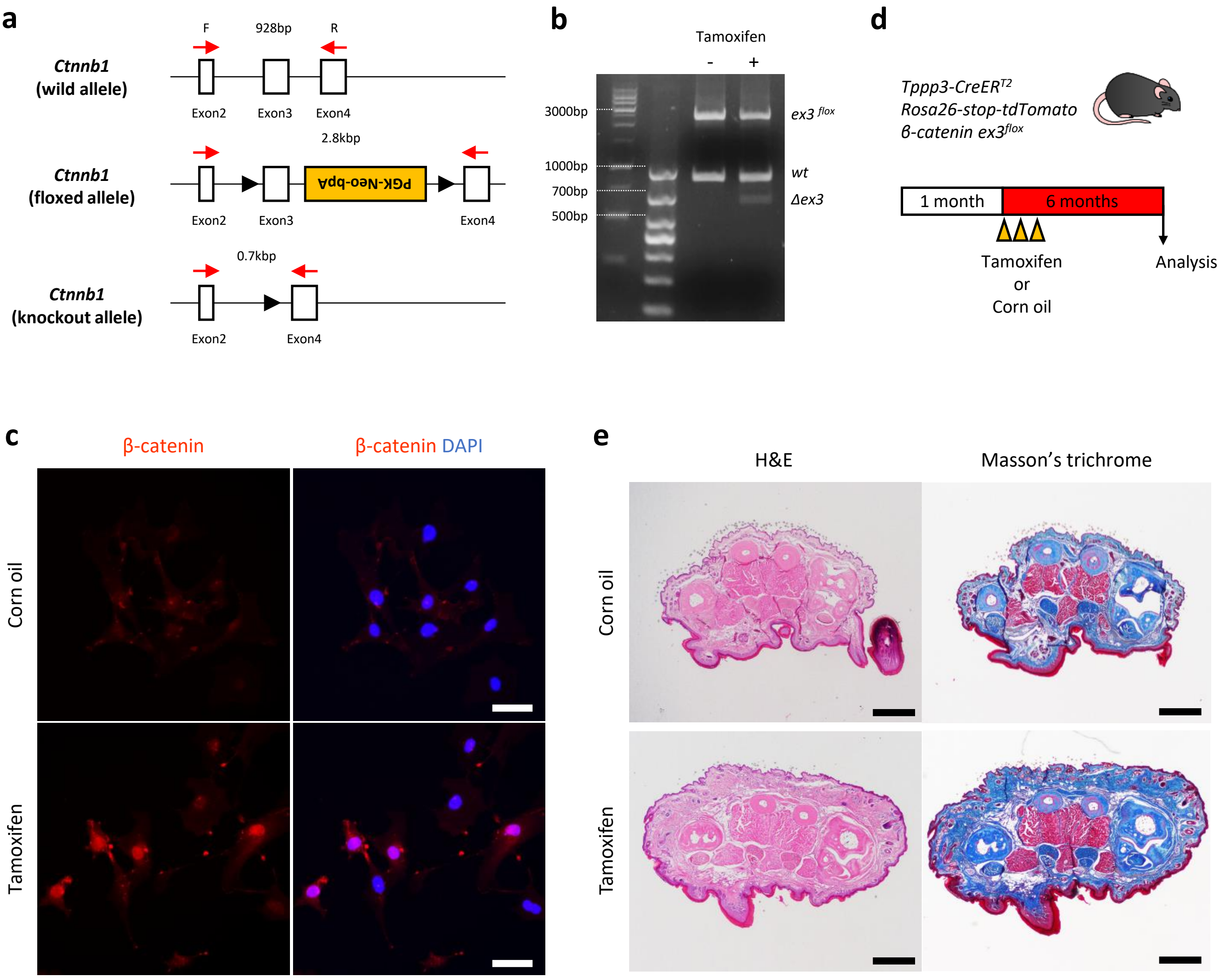


**Supplementary Fig. 2**

**Wnt/β-catenin signalling activation in *Tppp3*-positive cells induces dermal fibrosis in mouse paw.**

a. A schematic representation of *Ctnnb1* (*β-catenin*) wild, exon3 floxed (*ex3* $^{flox}$), and exon3 knockout (Δ*ex3*) alleles[61]. Red arrows indicate PCR primers. Primer sequences are as follows: forward (F), 5′- GCGTGGACAATGGCTACTCA-3′; reverse (R), 5′- TCAGCTCAGGAATTGCACGT-3′.

b. Genotyping PCR for *Tppp3-CreER*$^{T2}$*/β-catenin ex3* $^{flox}$ mice treated with or without tamoxifen. Genomic DNA was extracted from the dermal fibrotic tissue in the paw.

c. Immunocytochemical analysis of dermal fibrotic cells *in vitro.* Cultured cells were obtained from *Tppp3-CreER*$^{T2}$*/β-catenin ex3* $^{flox}$ mice treated with or without tamoxifen. Tamoxifen-treated cells show nuclear localization of β-catenin (red) (indicated by white arrows). Scale bars represent 50 µm.

d. A schematic representation of the *in vivo* experiment on *Tppp3-CreER*$^{T2}$*/R26-tdT/β-catenin ex3* $^{flox}$ mice. At 1 month of age, the mice were injected with 1 mg of tamoxifen or corn oil (control). After 6 months, their paws were analysed.

e. Haematoxylin and eosin (H&E) and Masson's trichrome staining of the paws in *Tppp3-CreER*$^{T2}$*/Rosa26-stop-tdTomato/β-catenin ex3* $^{flox}$ mice treated with tamoxifen or corn oil (control) (Fig. S2a). At 6 months after tamoxifen injection, fibrotic layers in the dermis were thickened in mice with β-catenin-activation (lower). Scale bars represent 400 µm.

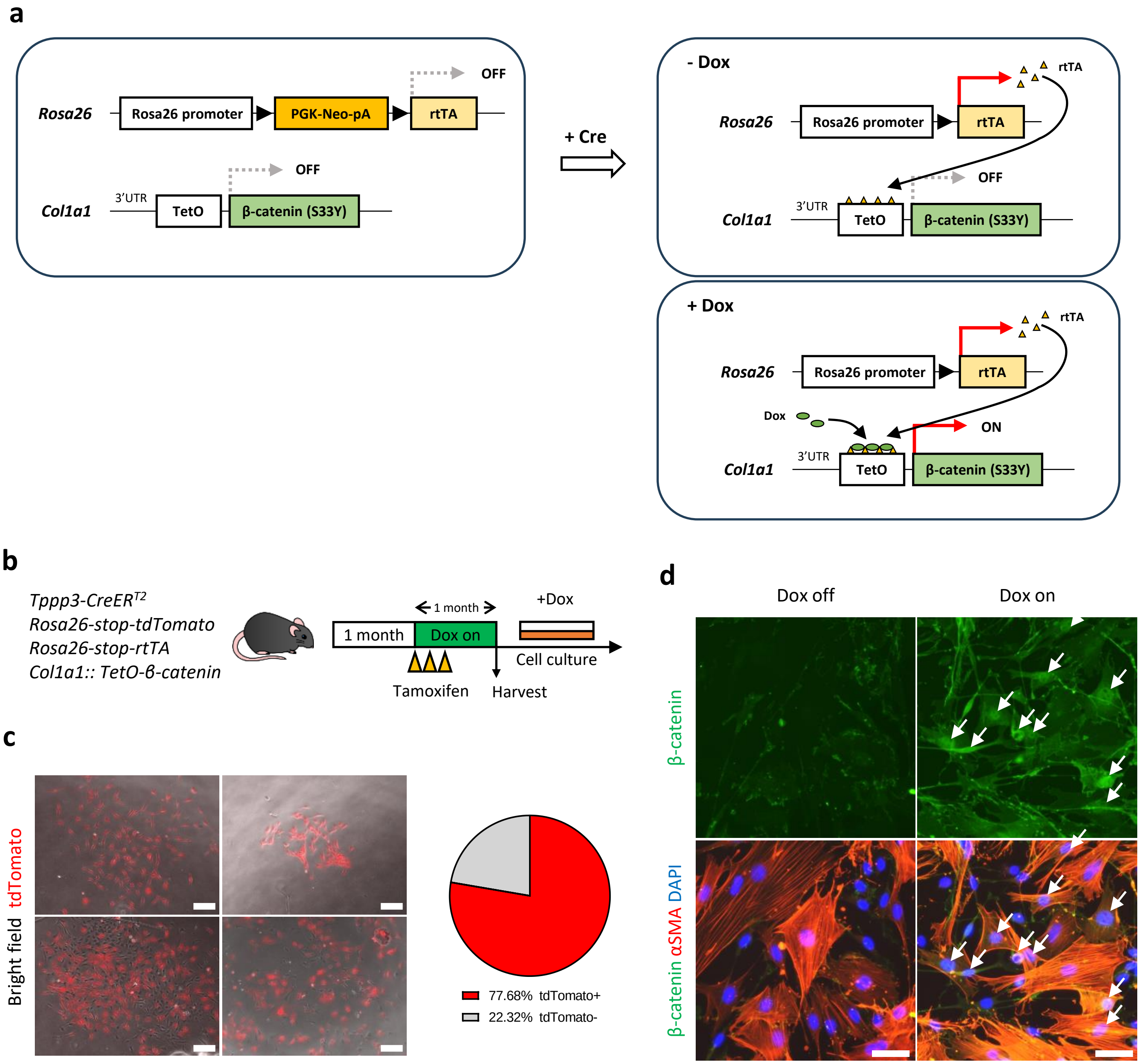


**Supplementary Fig. 3**

**Cell isolation from dermal fibrotic tissues in Dox-inducible β-catenin-expressing mice.**

a. A schematic of the *Rosa26-stop-rtTA/Col1a1:TetO-β-catenin* system. After Cre-recombination, reverse tetracycline transactivator (rtTA) is expressed from *Rosa26* loci. Subsequently, doxycycline (Dox) administration induces *β-catenin* expression from the TetO promoter in the 3' non-coding region of *Col1a1* loci.

b. A schematic of the cell isolation protocol from dermal fibrotic tissues in the forepaws of *Tppp3-CreER$^{T2}$/Rosa26-stop-tdTomato/Rosa26-stop-rtTA/Col1a1:TetO-β-catenin* mice.

c. Dermal fibrotic tissues were collected by removing skin, muscle, and tendons from the paws under a surgical microscope. Fibrotic cells were cultured with Dox-containing media (0.2 µg/mL). Fibrotic cells with passage 0 are shown. An average of 77.68% of cultured cells expressed tdTomato. Scale bars represent 200 µm.

d. Immunocytochemical analysis of the dermal fibrotic cells *in vitro.* Cultured cells were obtained from *Tppp3-CreER$^{T2}$/Rosa26-stop-rtTA/Col1a1:TetO-β-catenin* mice treated with tamoxifen. *β-catenin* expression was induced by Dox administration, and its nuclear localization was observed. Scale bars represent 50 µm.

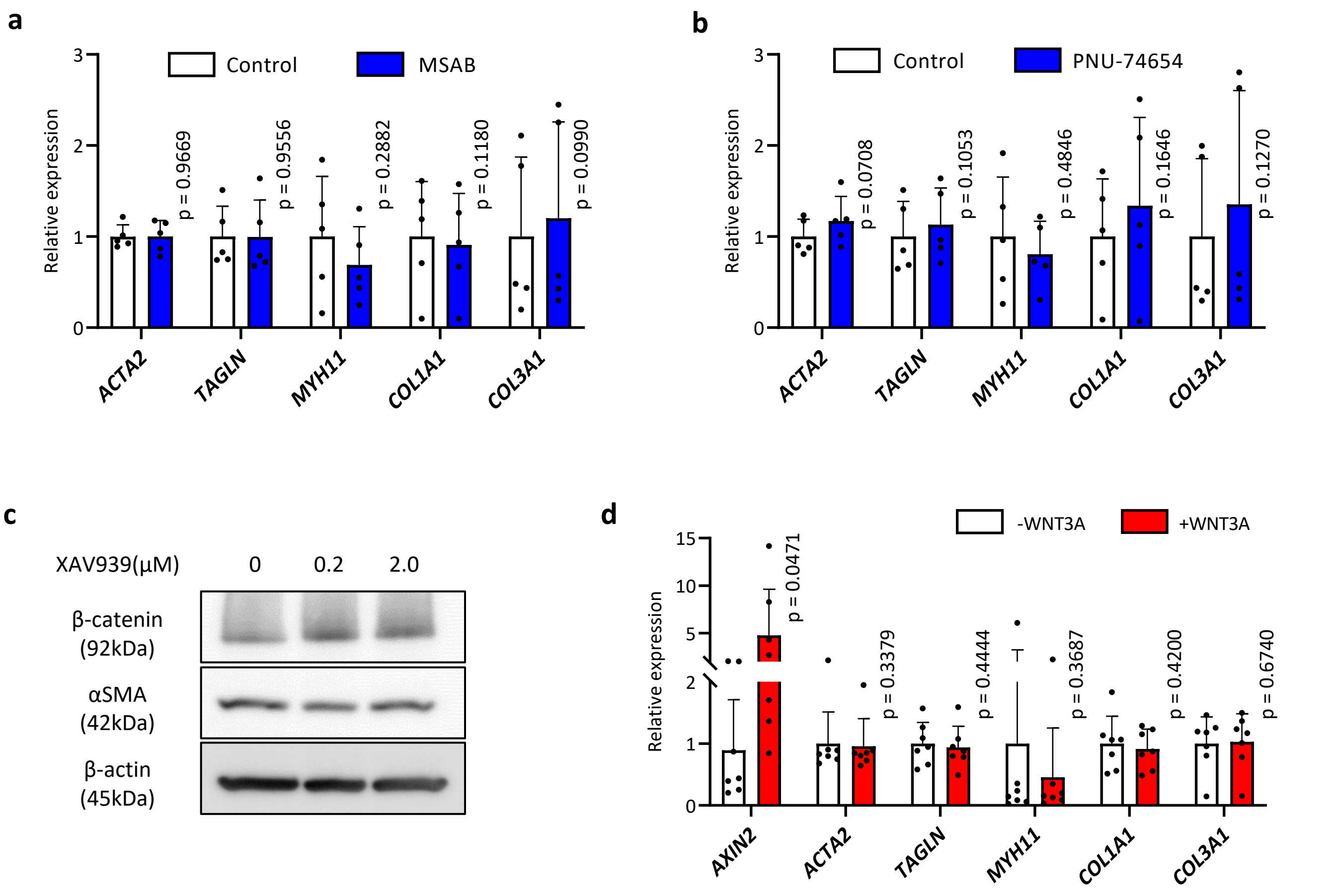


**Supplementary Fig. 4**

**Inhibition of Wnt/β-catenin signalling does not inhibit the myofibroblast phenotype and collagen production in human Dupuytren's contracture-derived cells.**

a. Real-time PCR results showing the expression of myofibroblast markers and collagen in human Dupuytren's contracture-derived cells treated with 1 μM of MSAB, a β-catenin inhibitor. The mean $\pm$ SD values of five independent biological samples (three technical replicates per sample) are shown. A two-tailed paired *t*-test was used for statistical analysis.

b. Real-time PCR results showing the expression of myofibroblast markers and collagen in human Dupuytren's contracture-derived cells treated with 25 μM of PNU-74654, an inhibitor of the β-catenin-TCF/LEF interaction. The mean $\pm$ SD values of five independent biological samples (three technical replicates per sample) are shown. A two-tailed paired *t*-test was used for statistical analysis.

c. Detection of β-catenin and αSMA expression in human Dupuytren's contracture-derived cells (DD-6) treated with XAV939 using western blotting. β-actin was used as an internal control.

d. Real-time PCR results of human Dupuytren's contracture-derived cells treated with 100 ng/mL of recombinant Wnt3A. The expression of a β-catenin target gene, *AXIN2*, was significantly upregulated following Wnt3A treatment ($p$ = 0.0471), whereas the expression of myofibroblast markers and collagens remained unchanged. The mean $\pm$ SD values of seven independent biological samples (three technical replicates per sample) are shown. A two-tailed paired *t*-test was used for statistical analysis.

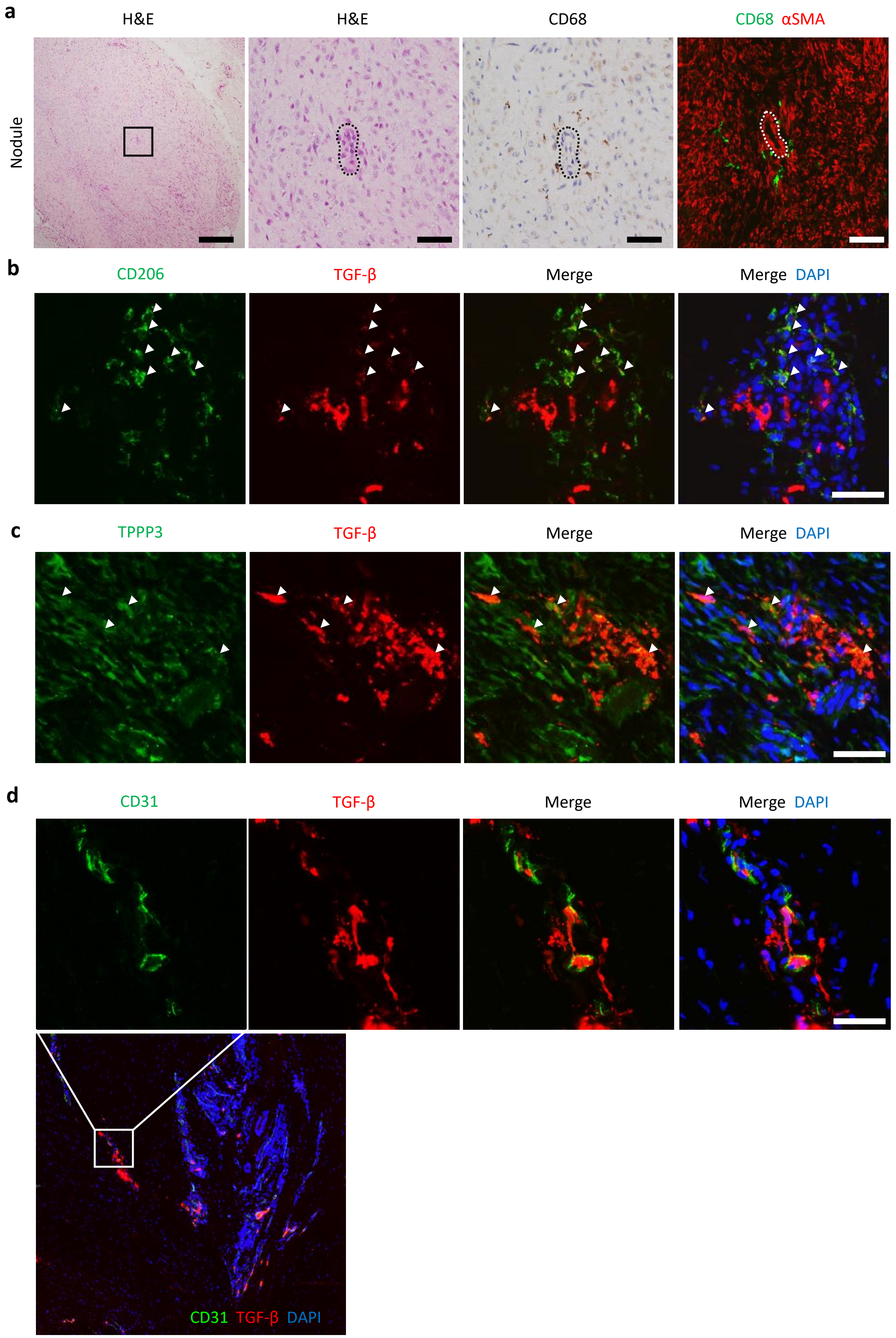

a
H&E
H&E
CD68
CD68 αSMA
Nodule
b
CD206
TGF-β
Merge
Merge DAPI
c
TPPP3
TGF-β
Merge
Merge DAPI
d
CD31
TGF-β
Merge
Merge DAPI
CD31 TGF-β DAPI

**Supplementary Fig. 5**

**The TGF-β expressing niche shows macrophage infiltration in human Dupuytren's contractures.**

a. Haematoxylin and eosin staining (H&E) and immunohistochemistry of the nodule of a human Dupuytren's contracture sample. CD68-expressing macrophages were present in the nodule composed of αSMA-expressing myofibroblasts. Macrophages were predominantly present around the vessel structure, as indicated by the dotted line.

b. Fluorescent immunohistochemistry of CD206 (green) and TGF-β (red) in the nodule of a human Dupuytren's contracture sample. TGF-β expression was observed in CD206-expressing macrophages (white arrowheads) and their adjacent cells.

c. Fluorescent immunohistochemistry of TPPP3 (green) and TGF-β (red) in the nodule of human Dupuytren's contracture tissue. TGF-β expression was detectable in TPPP3-expressing stromal cells (white arrowheads).

d. Fluorescent immunohistochemistry of CD31 (green) and TGF-β (red) in the nodule of human Dupuytren's contracture tissue. TGF-β expression was sparsely observed in the lower magnification panel. The white square area is enlarged in the upper panel. TGF-β expression was predominantly observed around vessel structures.

Scale bars represent 50 μm (a, b, d).

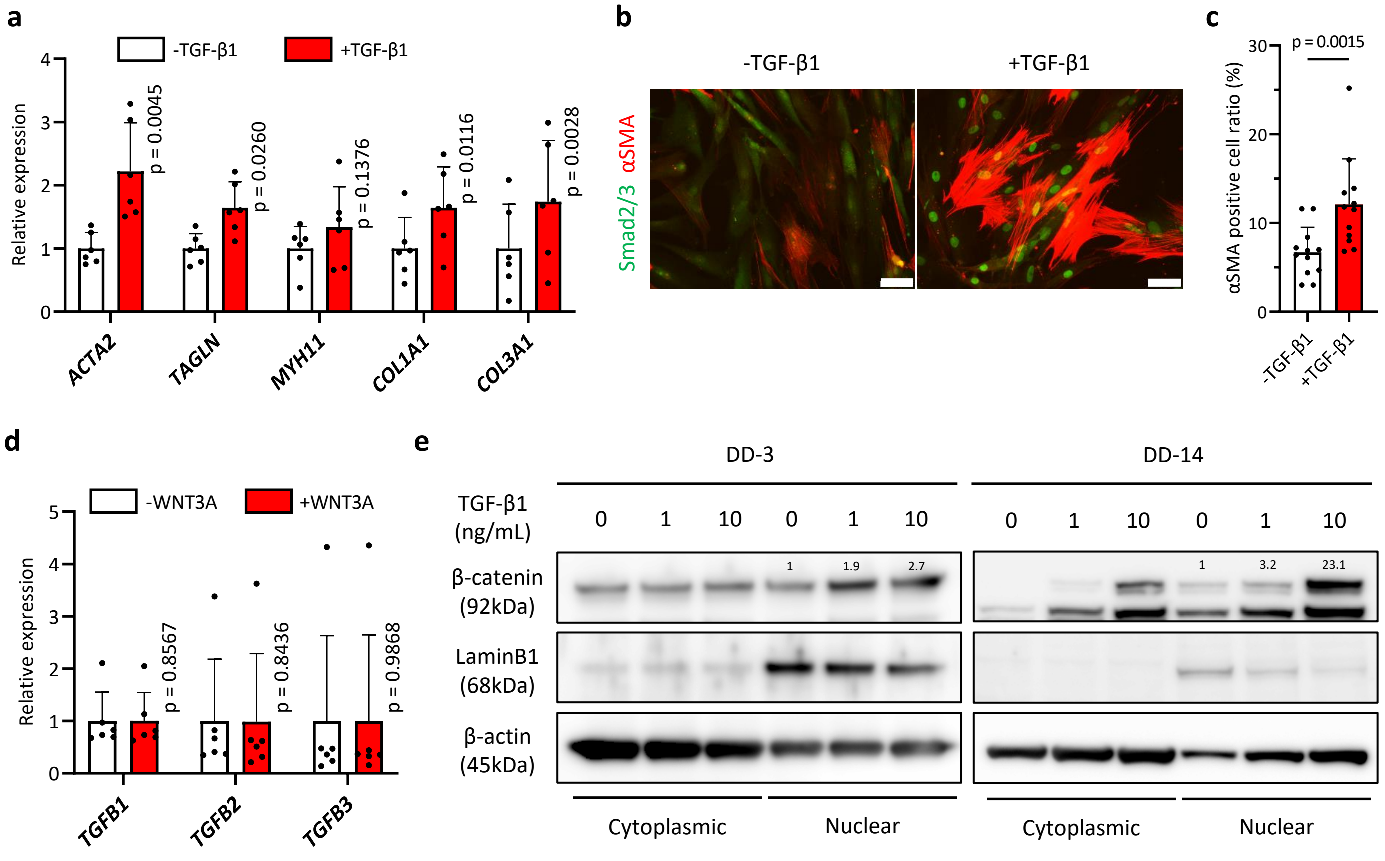


**Supplementary Fig. 6**

**TGF-β1 treatment induces fibrotic responses in human Dupuytren's contracture-derived cells.**

a. Gene expression analyses using real-time PCR of TGF-β1 (10 ng/mL)-treated human Dupuytren's contracture-derived cells *in vitro*. The mean ± SD values of six independent biological samples (three technical replicates per sample) are shown. A two-tailed paired *t*-test was used for statistical analysis.

b. Immunocytochemical analysis of TGF-β1 (10 ng/mL)-treated human Dupuytren's contracture-derived cells *in vitro*. TGF-β1 treatment induced nuclear localization of Smad2/3 (green) and αSMA-expressing myofibroblast differentiation (red) in Dupuytren's contracture-derived cells. Scale bars represent 100 µm.

c. αSMA-positive cell ratio of human Dupuytren's contracture-derived cells treated with or without TGF-β1 (10 ng/mL). TGF-β1 treatment significantly increased the number of αSMA-positive cells (n = 3; independent samples, 4 random different fields per n). The data are presented as the mean ± SD. A Mann–Whitney U-test was used for statistical analysis (p = 0.0015).

*d.* *TGFB1*, *TGFB2*, and *TGFB3* expression in recombinant Wnt3A (100 ng/mL)-treated human Dupuytren's contracture-derived cells, as analysed using real-time PCR. Activation of Wnt/β-catenin signalling induced no significant upregulation of *TGFB1, TGFB2*, and *TGFB3* expression (*p* = 0.8567, 0.8436, and 0.9868, respectively). The mean ± SD values of six independent biological samples (three technical replicates per sample) are shown. A two-tailed paired *t*-test was used for statistical analysis.

e. Detection of β-catenin expression in the nucleus and cytoplasm of human Dupuytren's contracture-derived cells via western blotting. Two independent samples (DD-3 and DD-14) were used. β-actin served as an internal control for cytoplasmic protein expression, whereas LaminB1 served as an internal control for nuclear protein expression. β-catenin/LaminB1 expression in the TGF-β1 0 ng/mL (control) group was set to 1, and β-catenin/LaminB1 expression in the TGF-β1 1 ng/mL and 10 ng/mL groups was measured. The results demonstrate that TGF-β1 treatment induced the nuclear translocation of β-catenin in both groups.

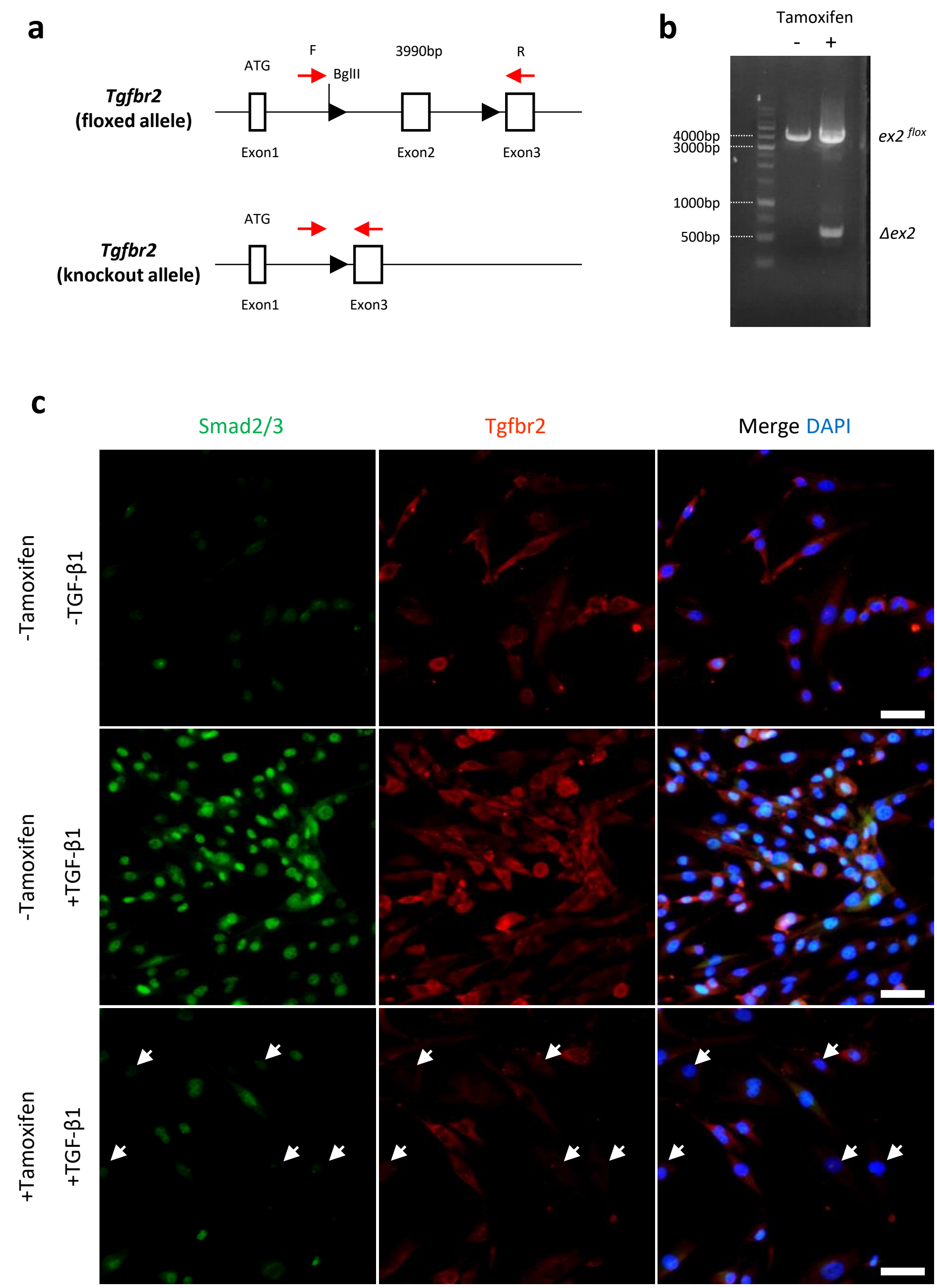


**Supplementary Fig. 7**

**Depletion of TGF-β signalling in *Tgfbr2* floxed mice.**

a. A schematic of *Tgfbr2* wild and exon2 floxed (*ex2* $^{flox}$) and exon2 knockout (Δ*ex2*) alleles[63]. Red arrows indicate PCR primers. Primer sequences are as follows: forward (F), 5′-TTAAACAAGGTCCGGAGCCC-3′; reverse (R), 5′- GAAGCGGCATCTTCCAGAGT-3′.

b. Genotyping PCR for *Tppp3-CreER*$^{T2}$*/Tgfbr2* $^{flox/flox}$ mice treated with or without tamoxifen. Genomic DNA was extracted from the dermal fibrotic tissue in the paw.

c. Immunocytochemical analysis of the dermal fibrotic cells *in vitro.* Cultured cells were obtained from *Tppp3-CreER*$^{T2}$*/Tgfbr2* $^{flox/flox}$ mice treated with or without tamoxifen. Tamoxifen-untreated dermal fibrotic cells expressing Tgfbr2 (red) showed strong nuclear localization of Smad2/3 (green) after TGF-β1 (10 ng/mL) treatment for 24 hours. Compared to those, tamoxifen-treated dermal fibrotic cells losing Tgfbr2 expression (indicated by white arrows) showed decreased nuclear localization of Smad2/3 after TGF-β1 treatment. Scale bars represent 50 µm.

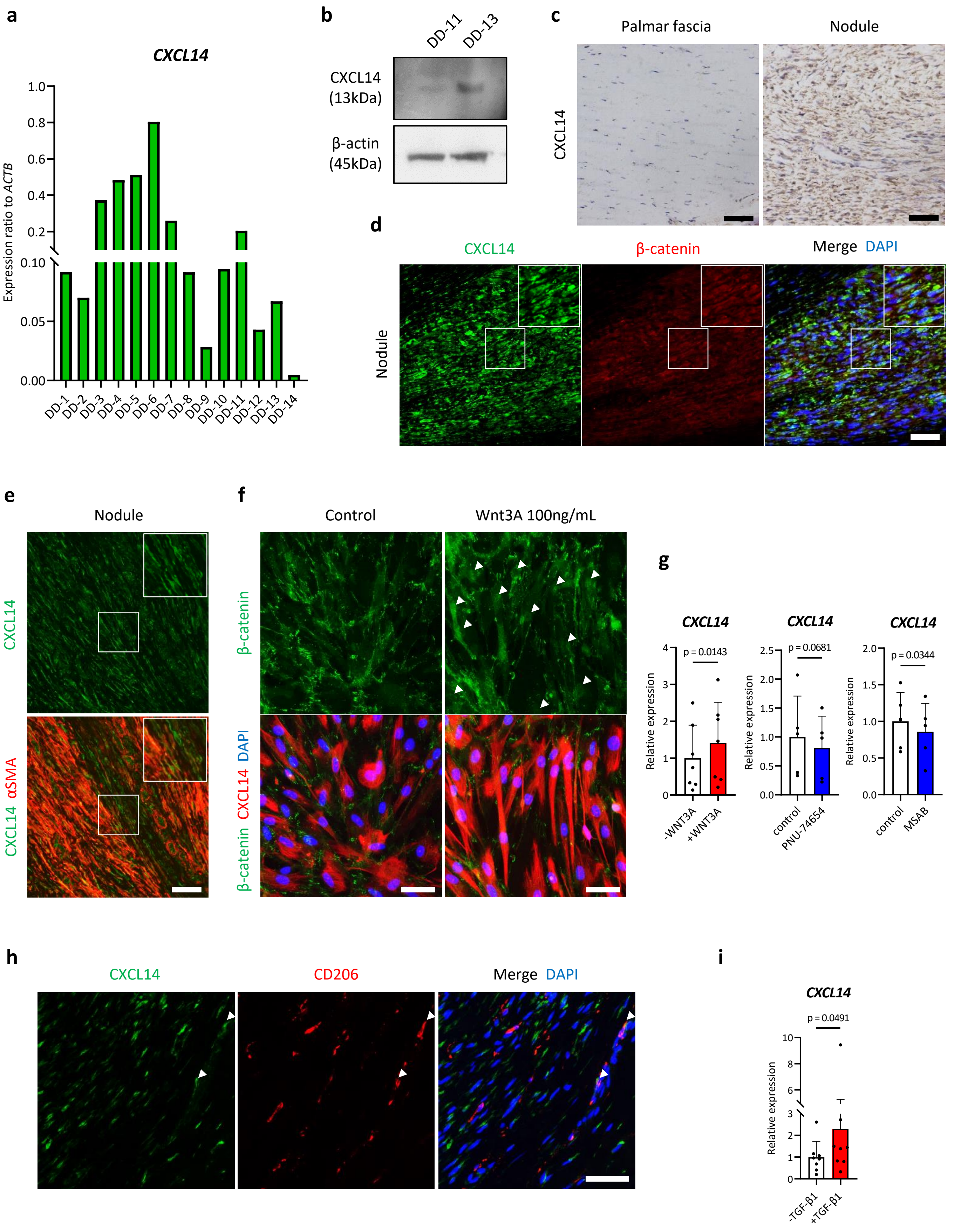
a
CXCL14
Expression ratio to ACTB
1.0
0.8
0.6
0.4
0.2
0.10
0.05
0.00
DD-1
DD-2
DD-3
DD-4
DD-5
DD-6
DD-7
DD-8
DD-9
DD-10
DD-11
DD-12
DD-13
DD-14
b
DD-11
DD-13
CXCL14
(13kDa)
β-actin
(45kDa)
c
Palmar fascia
Nodule
CXCL14
d
CXCL14
β-catenin
Merge DAPI
Nodule
e
Nodule
CXCL14
CXCL14 αSMA
f
Control
Wnt3A 100ng/mL
β-catenin
β-catenin CXCL14 DAPI
g
CXCL14
Relative expression
p = 0.0143
-WNT3A
+WNT3A
CXCL14
p = 0.0681
control
PNU-74654
CXCL14
p = 0.0344
control
MSAB
h
CXCL14
CD206
Merge DAPI
i
CXCL14
p = 0.0491
Relative expression
-TGF-β1
+TGF-β1

**Supplementary Fig. 8**

***CXCL14* expression in human Dupuytren's contractures.**

*a.* *CXCL14* expression (RT-qPCR) in the nodules of surgically excised human Dupuytren's contracture samples (DD-1 to DD-14) (also see Supplementary Fig. 1). The expression of *CXCL14* was normalized to *ACTB* expression. The mean of three technical replicates are shown.

b. Detection of CXCL14 expression in human Dupuytren's contracture samples (DD-11 and DD-13) using western blotting.

c. Immunohistochemistry of CXCL14 in the nodule of human Dupuytren's contracture tissues (upper) and normal palmar fascia tissues (lower) derived from the same patient. CXCL14 was strongly expressed in the nodule.

d. Fluorescent immunohistochemistry of CXCL14 (green) and β-catenin (red) in the nodule of human Dupuytren's contracture tissues. β-catenin-expressing cells co-expressed CXCL14. The white square area is enlarged in the upper right panel.

e. Fluorescent immunohistochemistry of CXCL14 (green) and α-SMA (red) in the nodule of human Dupuytren's contracture tissues. Both myofibroblasts (α-SMA-positive) and fibroblasts (α-SMA-negative) expressed CXCL14. The white square area is enlarged in the upper right panel.

f. Immunocytochemical analysis of Wnt3A (100 ng/mL)-treated human Dupuytren's contracture-derived cells *in vitro*. Wnt3A treatment induced nuclear localization (indicated by white arrowheads) of β-catenin (green) and upregulated CXCL14 (red) expression in Dupuytren's cells.

*g.* *CXCL14* expression (RT-qPCR) in recombinant Wnt3A (100 ng/mL) and Wnt signal inhibitor (PNU-74654 and MSAB)-treated human Dupuytren's contracture-derived cells. Wnt3A treatment significantly upregulated *CXCL14* expression ($p$ = 0.0143), whereas MSAB treatment significantly downregulated *CXCL14* expression ($p$ = 0.0344). The mean $\pm$ SD of seven and five independent biological samples for Wnt3A and Wnt inhibitor treatments (three technical replicates per sample) are shown, respectively. A two-tailed paired $t$-test was used for statistical analyses.

h. Fluorescent immunohistochemistry of CXCL14 (green) and CD206 (red) in a nodule of human Dupuytren's contracture tissues. Similar to that in murine fibrotic lesions, CD206-expressing macrophages were polarized around CXCL14-expressing cells. Some CD206-expressing macrophages co-expressed CXCL14 (indicated by white arrowheads).

*i.* *CXCL14* expression (RT-qPCR) in recombinant TGF-β1 (10 ng/mL)-treated human Dupuytren's contracture-derived cells. TGF-β1 treatment significantly upregulated *CXCL14* expression ($p$ = 0.0491). The mean $\pm$ SD of eight independent biological samples (three technical replicates per sample) are shown. A two-tailed paired $t$-test was used for statistical analyses.

Scale bars represent 50 µm (b, c, d, e, g).

## Fig. 1d

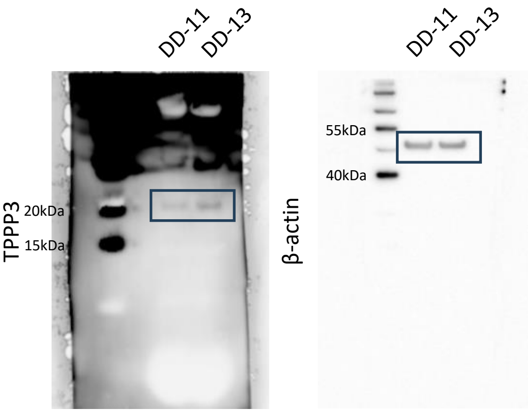


## Supplementary Fig. 4c

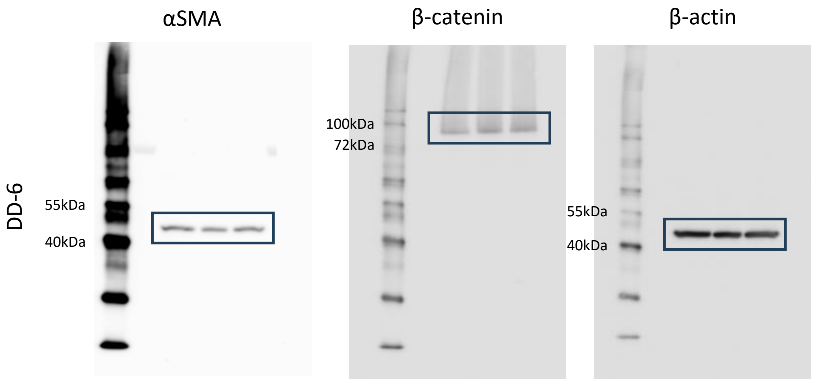


## Supplementary Fig. 2b

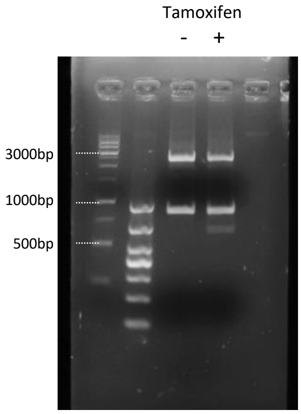


## Supplementary Fig. 6e

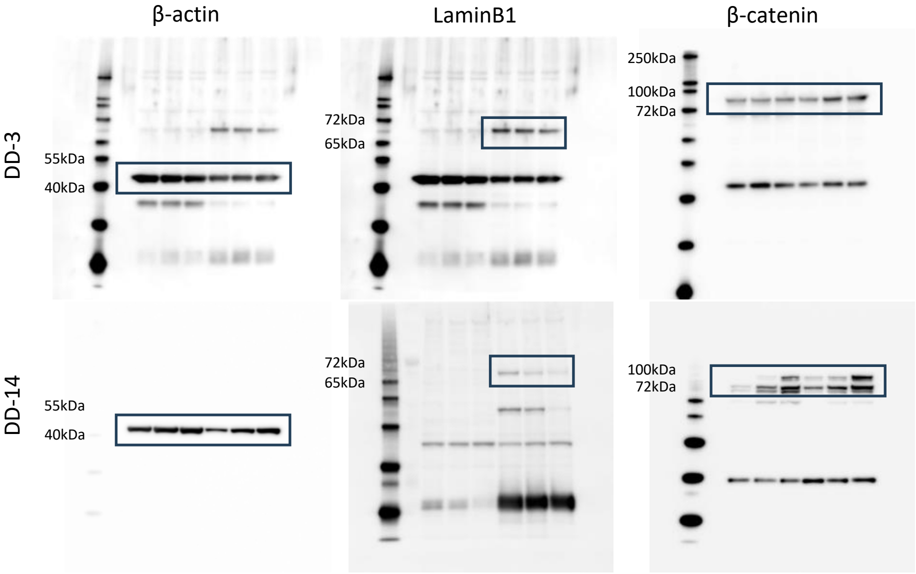


## Supplementary Fig. 7b

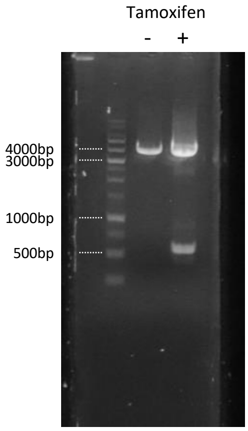


## Supplementary Fig. 8b

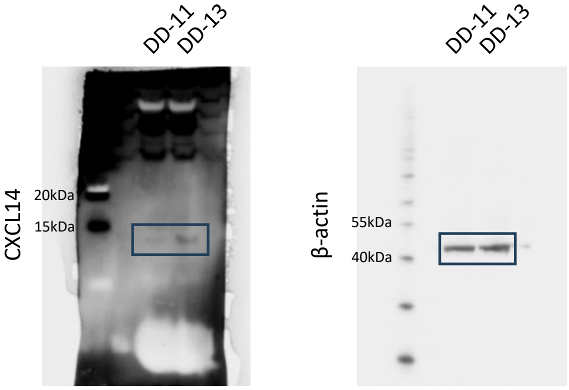


**Supplementary Fig. 9**
**Images of the full gel and membrane supporting Fig. 1d and Supplementary Fig. 2b, 4c, 6e, 7b and 8b.**

**Supplementary Table 1. Primer sequences used in this study**

| | | Genes | Forward (5' ⇒ 3') | Revers (5' ⇒ 3') |
|---|---|---|---|---|
| mouse | realtime PCR | *Acta2* | GTACCCAGGCATTGCTGACA | GAGGCGCTGATCCACAAAAC |
| | | *Tagln* | AACGATGGAAACTACCGTGGAG | TGCAGTTGGCTGTCTGTGAAG |
| | | *Myh11* | AGGCCAAGATTGCACAGCTG | GTCCTTTTGCTTCAGCGACTTG |
| | | *Col1a1* | TGGCGGTTATGACTTCAGCTTCCT | GGTCACGAACCACGTTAGCATCAT |
| | | *Col3a1* | CATAATGGGGAACGTGGTCCTC | CTGACCATCTGATCCAGGGTTTC |
| | | *Axin2* | ATGTCCTGTCTGCCAGCGTTC | CAAGCACTAGCCAGTGGGTCAA |
| | | *Tgfb1* | CAAGGGCTACCATGCCAACT | GTACTGTGTGTCCAGGCTCCAA |
| | | *Tgfb2* | GGGTACAATGCTAACTTCTGTGC | ATCCTGGGACACACAGCAAG |
| | | *Tgfb3* | GGGTTACTATGCCAACTTCTGC | TGGGTTCAGGGTGTTGTATAGTC |
| | | *Nos2* | GGCTACCACATTGAAGAAGCTG | TGCCCCATAGGAAAAGACTGC |
| | | *Tnfa* | AGCCTCTTCTCATTCCTGCTTG | TGATGAGAGGGAGGCCATTTG |
| | | *Mrc1* | TTTACGAGAAGTTGGGGTCAGG | AGCAGTCTCGATGGAAACCAG |
| | | *Arg1* | ATCAACACTCCCCTGACAACC | TTGGCAGATATGCAGGGAGTC |
| | | *Ccl2* | AGCAGGTGTCCCAAAGAAGC | ATTCCTTCTTGGGGTCAGCAC |
| | | *Ccl17* | TCAAAGGGGCCATTCCTATCAG | GTTTGTCTTTGGGGTCTGCAC |
| | | *Ccl22* | ATCTGCTGCCAGGACTACATC | ATCGGCACAGATATCTCGGTTC |
| | | *Cxcl6* | AAGGAGGTCTGTCTGGATCCAG | TGAACACTGGCCGTTCTTTCC |
| | | *Cxcl10* | GTGCTGCCGTCATTTTCTGC | CGCAGGGATGATTTCAAGCTTC |
| | | *Cxcl12* | TCCTCAACACTCCAAACTGTGC | TCGGGTCAATGCACACTTGTC |
| | | *Cxcl14* | TGAAGCCAAAGTACCCACACTG | CTTGATGAAGCGTTTGGTGCTC |
| | | *Actb* | GCCAACCGTGAAAAGATGAC | TCCGGAGTCCATCACAATG |
| human | realtime PCR | *TPPP3* | ATGGCAAGAACTGGGCCAAG | TTGATGACCCGAGCAGACTTC |
| | | *ACTA2* | ACCGAATGCAGAAGGAGATCAC | CCGATCCAGACAGAGTATTTGCG |
| | | *TAGLN* | TGGCAGTGACCAAGAATGATGG | AGGCCAATGACATGCTTTCCC |
| | | *MYH11* | TCCAAGTTCAAGTCCACCATCG | TCTTTCTGCTTCAGCGACTTGG |
| | | *COL1A1* | ACTGGTGAGACCTGCGTGTAC | TCGAACTGGAATCCATCGGTC |
| | | *COL3A1* | TGGGGAAACATGCATAAGTG | GCTAAACTGAAAACCACCATCC |
| | | *AXIN2* | TGTTCCGAACTTTCCTGGAG | GACAAAAGTCTTTGGCTACTCG |
| | | *TGFB1* | CCCTGCCCCTACATTTGGAG | CCCGGGTTATGCTGGTTGTAC |
| | | *TGFB2* | GGGTACAATGCCAACTTCTGTG | CAGCAAGGAGAAGCAGATGC |
| | | *TGFB3* | ACTATGCCAACTTCTGCTCAGG | AGATGCTTCAGGGTTCAGAGTG |
| | | *CXCL14* | AAGCCAAAGTACCCGCACTG | TACCACTTGATGAAGCGCTTGG |
| | | *ACTB* | AGACCTGTACGCCAACACAG | GGAGCAATGATCTTGATCTTCA |
