# Supplementary material for: C-X-C domain ligand 14-mediated stromal cell–macrophage interaction as a therapeutic target for hand dermal fibrosis
Source: Commun Biol. 2023 Nov 18;6:1173. doi: 10.1038/s42003-023-05558-8 (PMC10657354; doi:10.1038/s42003-023-05558-8)
Supplement: Supplementary file 2 — Description of Additional Supplementary Files [file 42003_2023_5558_MOESM2_ESM.docx]

Description of Additional Supplementary Files

**File name:** Supporting Data

**Description**: Supporting Data file and Futured Image file.
