# Supplementary material for: C-X-C domain ligand 14-mediated stromal cell–macrophage interaction as a therapeutic target for hand dermal fibrosis
Source: Commun Biol. 2023 Nov 18;6:1173. doi: 10.1038/s42003-023-05558-8 (PMC10657354; doi:10.1038/s42003-023-05558-8)
Supplement: Supplementary file 4 — Reporting Summary [file 42003_2023_5558_MOESM4_ESM.pdf]

nature portfolio

Corresponding author(s): Shingo Komura

Last updated by author(s): Nov 3, 2023

Reporting Summary

Nature Portfolio wishes to improve the reproducibility of the work that we publish. This form provides structure for consistency and transparency in reporting. For further information on Nature Portfolio policies, see our Editorial Policies and the Editorial Policy Checklist.

Statistics

For all statistical analyses, confirm that the following items are present in the figure legend, table legend, main text, or Methods section.

n/a	Confirmed	
☐	☒	The exact sample size (n) for each experimental group/condition, given as a discrete number and unit of measurement
☐	☒	A statement on whether measurements were taken from distinct samples or whether the same sample was measured repeatedly
☐	☒	The statistical test(s) used AND whether they are one- or two-sided<br>*Only common tests should be described solely by name; describe more complex techniques in the Methods section.*
☒	☐	A description of all covariates tested
☒	☐	A description of any assumptions or corrections, such as tests of normality and adjustment for multiple comparisons
☐	☒	A full description of the statistical parameters including central tendency (e.g. means) or other basic estimates (e.g. regression coefficient) AND variation (e.g. standard deviation) or associated estimates of uncertainty (e.g. confidence intervals)
☒	☐	For null hypothesis testing, the test statistic (e.g. F, t, r) with confidence intervals, effect sizes, degrees of freedom and P value noted<br>*Give P values as exact values whenever suitable.*
☒	☐	For Bayesian analysis, information on the choice of priors and Markov chain Monte Carlo settings
☒	☐	For hierarchical and complex designs, identification of the appropriate level for tests and full reporting of outcomes
☒	☐	Estimates of effect sizes (e.g. Cohen's d, Pearson's r), indicating how they were calculated

Our web collection on statistics for biologists contains articles on many of the points above.

Software and code

Policy information about availability of computer code

Data collection	GraphPad Prism 10.0.3
Data analysis	GraphPad Prism 10.0.3

For manuscripts utilizing custom algorithms or software that are central to the research but not yet described in published literature, software must be made available to editors and reviewers. We strongly encourage code deposition in a community repository (e.g. GitHub). See the Nature Portfolio guidelines for submitting code & software for further information.

Data

Policy information about availability of data

All manuscripts must include a data availability statement. This statement should provide the following information, where applicable:

- Accession codes, unique identifiers, or web links for publicly available datasets
- A description of any restrictions on data availability
- For clinical datasets or third party data, please ensure that the statement adheres to our policy

All data generated or analysed during this study are included in this published article (and its supplementary information files).

Human research participants

Policy information about studies involving human research participants and Sex and Gender in Research.

Reporting on sex and gender	DD-1: 72-year-old-femal, DD-2: 86-year-old male, DD-3: 76-year-old male, DD-4: 76-year-old male, DD-5: 71-year-old male, DD-6: 75-year-old male, DD-7: 51-year-old male, DD-8: 80-year-old male, DD-9: 71-year-old male, DD-10: 86-year-old male, DD-11: 75-year-old male, DD-12: 70-year-old male, DD-13: 79-year-old male, and DD-14: 55-year-old male.
Population characteristics	mentioned above
Recruitment	The patients underwent surgery for Dupuytren's contracture in Gifu University hospital were informed this research. We obtained informed consent from 10 patients who agreed with this research, and collected excised samples at the operation.
Ethics oversight	The Ethics Committee of Gifu University (Approval Number 28-140)

Note that full information on the approval of the study protocol must also be provided in the manuscript.

Field-specific reporting

Please select the one below that is the best fit for your research. If you are not sure, read the appropriate sections before making your selection.

☒ Life sciences ☐ Behavioural & social sciences ☐ Ecological, evolutionary & environmental sciences

For a reference copy of the document with all sections, see nature.com/documents/nr-reporting-summary-flat.pdf

Life sciences study design

All studies must disclose on these points even when the disclosure is negative.

Sample size	Sample size is described in methods section, Figures or Figure legends.<br>All real time-quantitative PCR experiments were performed in technical triplicate for each sample. In human Dupuytren's contracture tissue, TPPP3 and CXCL14 expression was measured 14 independent samples (Fig.1c and S8a). In human Dupuytren's contracture-derived cells 5-8 independent samples were analyzed in (Fig.S4a, S4b, S4d, S6a, S6d, S8g and S8i). In murine dermal fibrotic cells (Tppp3-CreERT2/Rosa26-stop-tdTomato/Rosa26-stop-rtTA/Col1a1::TetO-β-catenin), 7 independent samples were analyzed in (Fig.3e and 6a), and 6 independent samples were analyzed in (Fig.4j). In murine dermal fibrotic cells (Tppp3-CreERT2/β-catenin ex3 flox), 5 independent experiments were performed in (Fig. 4h and 6i). In murine RAW264.7, 3 and 7 independent experiments were performed in (Fig. 4e) and (Fig. 6d), respectively.<br>Immunocytochemistry was performed using murine dermal fibrotic cells (Tppp3-CreERT2/Rosa26-stop-tdTomato/Rosa26-stop-rtTA/Col1a1::TetO-β-catenin and Tppp3-CreERT2/β-catenin ex3 flox) and human Dupuytren's contracture-derived cells. The 3 independent samples (Fig.3f and 3g), 1 sample (3 independent experiments) (Fig.4i), 3 independent samples (Fig.S6b and S6c) were used for the analyses.<br>In cell migration and chemotaxis assay, RAW 267.4 cell line was used. The 3 independent experiments were performed in migration assay (Fig. 6e), and 4 and 3 independent experiments were performed in chemotaxis assay (Fig.6f and 6g, respectively).<br>In vivo analyses, 4-5 independent biological samples (6 different lesion per sample) were analyzed in (Fig.2e), 4 independent biological samples (2 different lesion per sample) were analyzed in (Fig.2f),6-8 independent biological samples (6 different lesion per sample) were analyzed in (Fig.3b), 6-8 independent biological samples (2 different lesion per sample) were analyzed in (Fig.3c), and 3 independent biological samples (2 different lesion per sample) were analyzed in (Fig.4d), 5-7 independent biological samples (6 different lesion per sample) were analyzed in (Fig.5b), 5-7 independent biological samples (2 different lesion per sample) were analyzed in (Fig.5c). In Fig. 7, 2 different regions of 4 independent samples per each group (Corn oil, Tamoxifen+IgG, Tamoxifen+Cxcl14 Ab) were analyzed in (Fig.7b and 7d). And, 4 different region of 5, 8 and 8 independent samples per Corn oil, Tamoxifen+IgG and Tamoxifen+Cxcl14 Ab group were analyzed in (Fig.7c).
Data exclusions	We excluded mice (n=4) which unexpectedly died during doxycycline administration. We didn't excluded any other data from the analysis to avoid the arbitrary selection.
Replication	Repeating experiments revealed that our data are reproducible. See methods section and each Figure legends.
Randomization	All in vitro and in vivo samples (murine dermal fibrotic cells, human Dupuytren's contracture-derived cells and mice) were allocated at random.
Blinding	Blinding measurement was performed for in vivo analyses (Fig.2, Fig. 3, Fig.5 and Fig.7).

Reporting for specific materials, systems and methods

We require information from authors about some types of materials, experimental systems and methods used in many studies. Here, indicate whether each material, system or method listed is relevant to your study. If you are not sure if a list item applies to your research, read the appropriate section before selecting a response.

Materials & experimental systems

n/a	Involved in the study
☐	☒ Antibodies
☒	☐ Eukaryotic cell lines
☐	☐ Palaeontology and archaeology
☐	☒ Animals and other organisms
☐	☒ Clinical data
☒	☐ Dual use research of concern

Methods

n/a	Involved in the study
☒	☐ ChIP-seq
☒	☐ Flow cytometry
☒	☐ MRI-based neuroimaging

Antibodies

Antibodies used	All supplier names, catalog numbers, clone names of primary antibodies which we used in this study were described in methods section.
Validation	Validation of dilution was determined in accordance with manufacturer's directions. In some primary antibodies, we optimized the dilution to fit our staining protocols.

Palaeontology and Archaeology

Specimen provenance	*Provide provenance information for specimens and describe permits that were obtained for the work (including the name of the issuing authority, the date of issue, and any identifying information). Permits should encompass collection and, where applicable, export.*
Specimen deposition	*Indicate where the specimens have been deposited to permit free access by other researchers.*
Dating methods	*If new dates are provided, describe how they were obtained (e.g. collection, storage, sample pretreatment and measurement), where they were obtained (i.e. lab name), the calibration program and the protocol for quality assurance OR state that no new dates are provided.*

☐ Tick this box to confirm that the raw and calibrated dates are available in the paper or in Supplementary Information.

Ethics oversight	*Identify the organization(s) that approved or provided guidance on the study protocol, OR state that no ethical approval or guidance was required and explain why not.*

Note that full information on the approval of the study protocol must also be provided in the manuscript.

Animals and other research organisms

Policy information about studies involving animals; ARRIVE guidelines recommended for reporting animal research, and Sex and Gender in Research

Laboratory animals	All genetically modified mice were maintained in C57/BL6 background. Mice lines used in this study were available in Methods section.
Wild animals	N/A
Reporting on sex	Both male and female mice were randomly used without bias.
Field-collected samples	N/A
Ethics oversight	All animal experiments were approved by the Gifu University Animal Experiment Committee (approval number 2019-183, 2021-094, 2021-238)

Note that full information on the approval of the study protocol must also be provided in the manuscript.

Clinical data

Policy information about clinical studies

All manuscripts should comply with the ICMJE guidelines for publication of clinical research and a completed CONSORT checklist must be included with all submissions.

Clinical trial registration	*Provide the trial registration number from ClinicalTrials.gov or an equivalent agency.*
Study protocol	*Note where the full trial protocol can be accessed OR if not available, explain why.*
Data collection	*Describe the settings and locales of data collection, noting the time periods of recruitment and data collection.*

Outcomes

Describe how you pre-defined primary and secondary outcome measures and how you assessed these measures.
